# Fibronectin1‐Expressing Subicular Circuits Selectively Govern the Retrieval of Novel Object Recognition

**DOI:** 10.1002/advs.202516399

**Published:** 2026-04-15

**Authors:** Fan Fei, Jiaying Shi, Jing Xi, Yixiang Xu, Shuye Ying, Xukun Fan, Wangjialu Lu, Zhisheng Li, Menghan Li, Yu Wang, Li Cheng, Lin Yang, Lingyu Xu, Zhong Chen, Cenglin Xu, Yi Wang

**Affiliations:** ^1^ Zhejiang Collaborative Innovation Center for the Brain Diseases with Integrative Medicine Zhejiang Key Laboratory of Neuropsychopharmacology School of Pharmaceutical Sciences & First Affiliated Hospital Zhejiang Chinese Medical University Hangzhou China; ^2^ Institute of Pharmacology and Toxicology College of Pharmaceutical Sciences School of Medicine Zhejiang University Hangzhou China

**Keywords:** entorhinal cortex, fibronectin1, neural circuit, novel object recognition, subiculum

## Abstract

Novel object recognition (NOR), referring to the cognitive ability to differentiate familiar/novel objects, is a fundamental cognitive function essential for daily life. However, the mechanisms underlying the encoding of novel preference information remain incompletely understood. Here, we demonstrate that the fibronectin1‐expressing subiculum–entorhinal circuits selectively govern NOR retrieval. Subicular pyramidal neurons are able to differentiate between familiar and novel objects, and bidirectionally regulate the retrieval of NOR. At the circuit level, subicular–entorhinal projections, rather than those targeting the anterior nucleus of thalamus, mammillary bodies or retrosplenial cortex, encode novel object preference and regulate NOR retrieval. Importantly, fibronectin1 is identified as a key functional molecular component expressing within this glutamatergic circuit, gates neuronal excitability by regulating the large conductance Ca^2+^‐activated potassium channel, and selectively governs the NOR retrieval. These findings provide novel insights into the molecular and circuit‐level mechanisms of NOR and highlight potential therapeutic targets for cognitive disorders targeting fibronectin1‐expressing subicular circuits.

## Graphical abstract

Fibronectin 1 (FN1)‐expressing subicular subpopulations encode novel object preference and selectively govern retrieval of novel object recognition (NOR) via affecting excitability of entorhinal‐projecting circuit through large conductance Ca2+‐activated potassium (BK) channel.

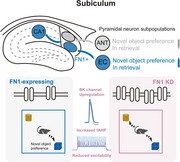

## 1 Introduction

Novel object recognition (NOR) memory refers to the cognitive ability to determine whether we have previously encountered a particular stimulus and to distinguish it from novel ones [1, 2, 3]. NOR plays a crucial role in everyday life, supporting functions ranging from recognizing faces to recalling past interactions [4]. Previous studies have highlighted the hippocampus and perirhinal cortex as key brain regions involved in NOR, with distinct contributions to recollection and familiarity, respectively [5, 6]. However, how novel preference information is encoded and coordinated between the hippocampus and the cortex remains incompletely understood. Specifically, within the critical hippocampal circuitry, most studies have focused on the cornu ammonis 1 (CA1) and dentate gyrus (DG) subregions [6, 7, 8], which are central components of the canonical ‘trisynaptic’ pathway, and have been widely implicated in the involvement of memory/cognition processing, while less attention is given to the subiculum, the primary output subregion of the hippocampus.

The subiculum shows important functions in navigation and memory, some of which may be independent of hippocampal function [9, 10, 11, 12]. Glutamatergic projecting from the subiculum to the entorhinal cortex (EC) specifically mediates the retrieval of fear condition memory, while in consolidation period, an enhanced connection between the subiculum and amygdala is found [13, 14]. Meanwhile, the subiculum is also reported to be involved in the object location learning, through its noncanonical pathway to the CA1 [15]. Moreover, theta power in the subiculum increased greatly when mice exploring a novel object than a familiar object, which is not the case in the CA1 [16]. These findings highlight functional diversity in cognition among subicular pyramidal neurons based on their projecting targets. Single cell RNA‐sequencing has further revealed pronounced molecular heterogeneity within subicular pyramidal neurons, identifying discrete subpopulations with distinct molecular markers and projection patterns [17, 18, 19]. However, the precise molecular mechanisms governing circuit‐specific regulation of NOR in subicular pyramidal neurons remain largely unexplored.

Here, we demonstrate that subicular pyramidal neurons specifically mediate NOR retrieval through projections to the EC, rather than the anterior nucleus of thalamus (ANT), the mammillary bodies (MMB), or the retrosplenial cortex (RSC). We further identify fibronectin 1 (FN1)—a key component of the extracellular matrix and a molecular marker specifically expressed within the subiculum—as a novel functional regulator enriched in this circuit. FN1 gates neuronal excitability by regulating BK potassium channel expression, thereby selectively governing NOR retrieval. These findings provide mechanistic insight into how FN1‐expressing subicular circuits selectively regulate NOR, and suggest potential therapeutic targets for cognitive disorders.

## 2 Results

### 2.1 Subicular Pyramidal Neurons Mediate the NOR Retrieval

**FIGURE 1:**
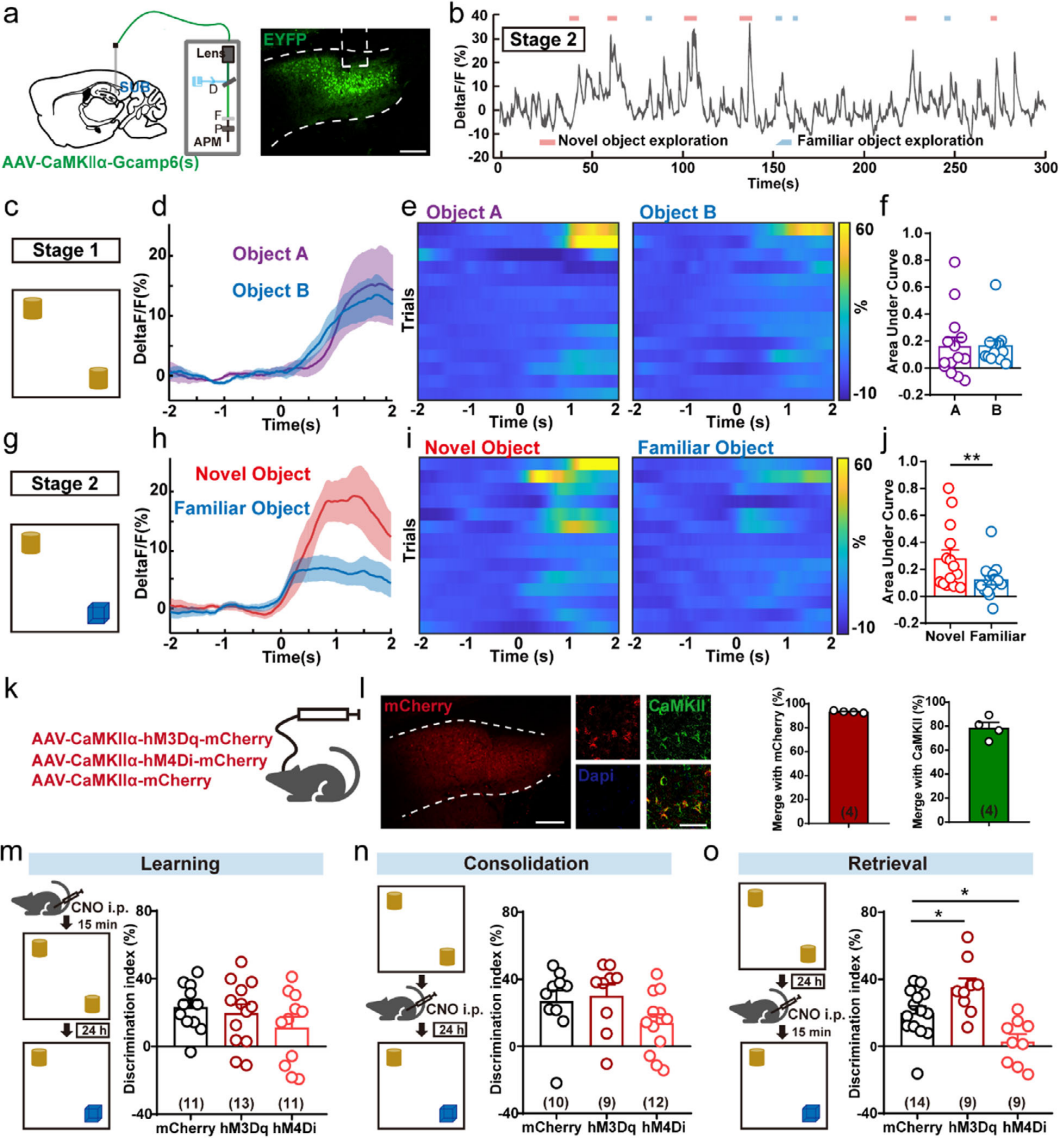
Subicular pyramidal neurons mediate novel object recognition retrieval. (a) Schematic of fiber photometry and representative image of GCaMP6(s) expression in the subiculum. Scale bar, 200 µm. (b) Representative changes in GCaMP6(s) fluorescence of subicular pyramidal neurons in the novel object recognition (NOR) test. (c–e) Averaged values (d) and heatmap (e) of GCaMP6(s) fluorescence of subicular pyramidal neurons during learning period of the NOR test. (f) Area under curve (AUC) of both delta F/F lines in (d). (g–i) Averaged values (h) and heatmap (i) of GCaMP6 (s) fluorescence of subicular pyramidal neurons during retrieval period of the NOR test. *N* = 7 mice, each mouse included 2 trials. (j) AUC of both delta F/F lines in (h). Paired *t* test, ** *p* < 0.01. k Schematic of viral injection in three different groups. (l) Representative images and quantification of mCherry expression in the subiculum. Scale bar, 200 µm. (m–o) Discrimination indexin the NOR test by chemogenetic activation/inhibition of subicular pyramidal neurons during learning (m), consolidation (n), or retrieval (o) period. One‐way ANOVA with post hoc Dunnett's multiple comparisons, *p < 0.05. The number of mice is indicated in the figure. Data are presented as mean± SEM.

We first evaluated the real‐time neural dynamics of subicular pyramidal neurons during different stages of NOR. This was accomplished by intrasubicular injection of AAV‐CaMKIIα‐GCaMP6s virus and subsequent calcium fiber photometry recording [20] (Figure 1a). Behavioral test compromised two stages, during the first sample stage, we observed that when mice explored two identical objects (same size, color, and shape), the calcium fluorescence of subicular pyramidal neurons both immediately and sharply increased with similar amplitude (Figure 1c–f, area under curve (AUC), object A 0.1619 ± 0.066 vs. object B 0.1621 ± 0.038, *p* = 0.9959), indicating they were able to respond to object exploration. In the second test stage, one familiar object was replaced with a novel object (same size, different color and shape). As shown in the representative example in Figure 1b, the increase in calcium fluorescence was significantly greater when the mice explored the novel object than when they explored the familiar one (Figure 1g–j, AUC, novel object 0.2801 ± 0.064 vs. familiar object 0.1191 ± 0.034, *p* = 0.0031). We performed a linear regression analysis between the AUC of the calcium signals and the fluorescence intensity of viral expression, and found that higher levels of GCaMP expression in the subiculum were associated with larger AUC values during the rising phase of the calcium signal. In addition, we compared the mice's overall locomotor speed in the open‐field test with their average speed during object exploration and found no significant difference between the two (Figure S1a,b), suggesting that the observed changes in calcium signals were not driven by concurrent alterations in locomotion. These above results demonstrate that subicular pyramidal neurons positively encode the novel object preference.

Cognitive memory is a complex process, which can be further divided into three phases: learning, consolidation, and retrieval [21]. It is generally believed that the stage 1 mainly refers to the learning period, and 6 h after is the start of consolidation period. To establish causal relationships, we used chemogenetics to modulate subicular activity during distinct memory phases. We first expressed hM3Dq or hM4Di in subicular pyramidal neurons and quantified the efficacy and accuracy of viral expression (Figure 1k,l mCherry group: 78.56 ± 4.64% of pyramidal neurons expressed mCherry and 93.27 ± 0.43% mCherry+ neurons were CaMKII positive). To investigate whether subicular pyramidal neurons are involved in the learning period of NOR memory, we administered clozapine N‐oxide (CNO) 15 min prior to the first stage. Mice subsequently underwent the retrieval task 24 h later. The results showed that manipulating subicular pyramidal neurons during the learning phase did not affect NOR memory (Figure 1m). To further examine their role in the consolidation period, CNO was administered 6 h after the initial learning task, followed by testing 18 h later. Similarly, intervention during the consolidation period did not alter NOR memory (Figure 1n). Finally, to determine whether these neurons contribute to the retrieval period, CNO was administered 15 min before the second stage. The results indicated activation of subicular pyramidal neurons enhanced, whereas inhibition suppressed, the retrieval of NOR memory (Figure 1o, mCherry 20.01 ± 3.93% vs. hM3Dq 35.10 ± 5.40%, *p* = 0.0448; mCherry vs. hM4Di, 2.828 ± 4.47%, *p* = 0.0211). We likewise performed a linear regression analysis between the discrimination index in the NOR task and the fluorescence intensity of viral expression. The results showed that higher levels of mCherry expression in the subiculum were associated with a more pronounced increase or decrease in discrimination index, depending on the direction of the manipulation (Figure S1c,d). In addition, we applied the same approach to examine the role of subicular pyramidal neurons during the retrieval phase of the object location recognition (OLR) task. The results showed that chemogenetic inhibition of subicular pyramidal neurons only had a slight tendency to reduce discrimination index (Figure S2, mCherry 30.94 ± 5.05% vs. hM4Di 19.88 ± 5.72%, *p* = 0.1664). These results demonstrate that subicular pyramidal neurons are specifically involved in the NOR retrieval.

### 2.2 The Subicular–Entorhinal Circuit Selectively Governs NOR Retrieval

Subicular pyramidal neurons project extensively to both cortical and subcortical regions, with the EC, ANT, and MMB being particularly implicated in cognitive processes [22]. To investigate their involvement in circuitry level, we recorded calcium signaling fluorescence from these three projection sites in mice during the second stage of the NOR task. A genetically engineered AAV‐EF1α‐DIO‐Axon‐GCaMP6(s) virus was accordingly injected into the subiculum of *CaMKIIα‐Cre* mice, and optical cannulas were implanted into the EC, ANT, and MMB to monitor calcium dynamics at the projection terminals (Figure S3a). During the test stage, fluorescence at the EC terminals exhibited a detectable increase when mice explored novel objects, much higher Ca^2+^ level than that of familiar object, suggesting subicular–entorhinal circuits may encode the novel object preference. Whereas no significant Ca^2+^ differences were observed at the ANT or MMB terminals when mice interacted with the novel or familiar object Figure S3b–g). These findings suggest that the subicular–entorhinal cortex circuits may play a specific role in processing novel object stimuli.

**FIGURE 2:**
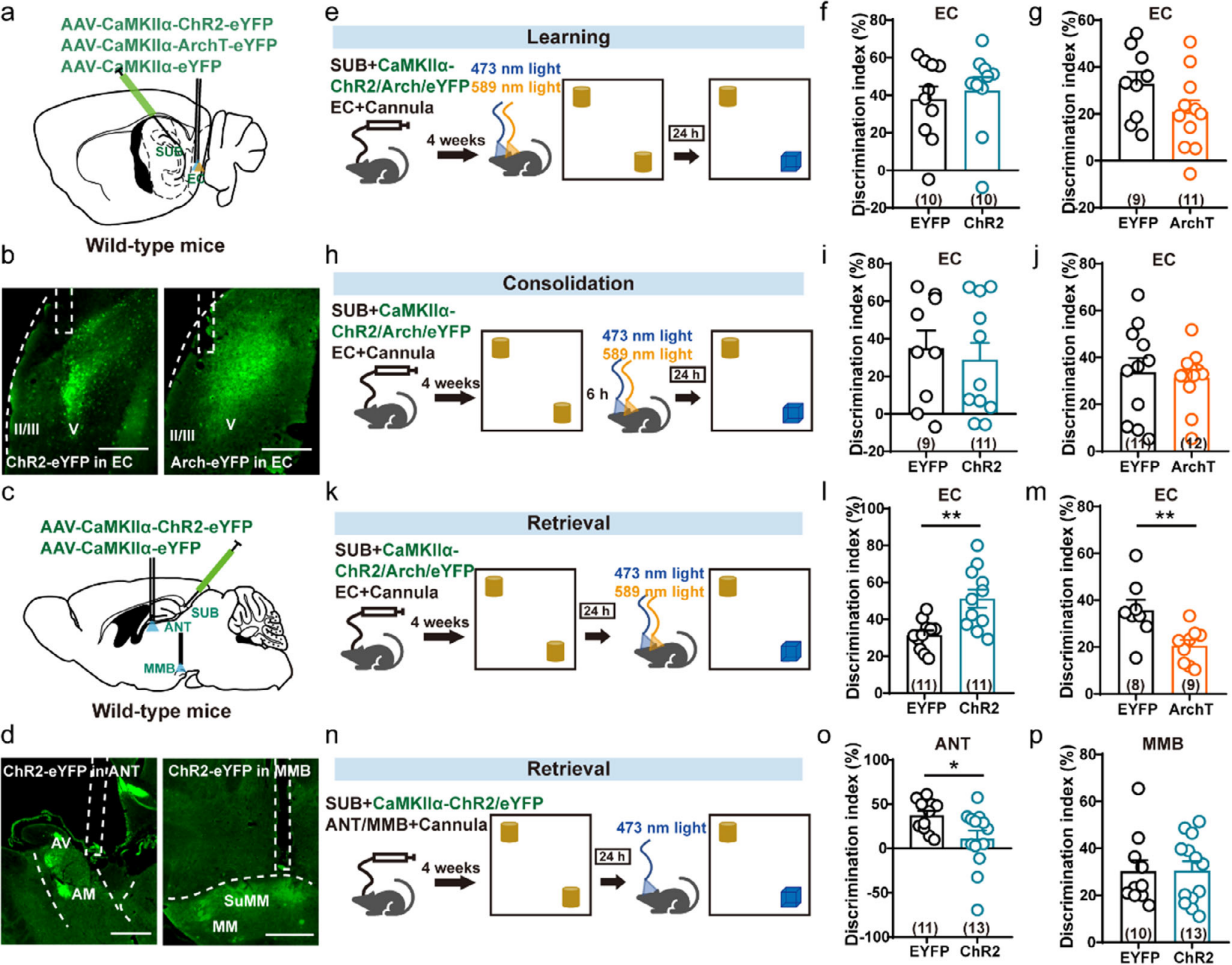
The subicular–entorhinal circuit selectively governs novel object recognition retrieval. (a–d) Schematic of viral injection in different groups and representative images of viral expression and cannula placement in the entorhinal cortex (EC), anterior nucleus of thalamus (ANT), and mammillary bodies (MMB). Scale bar, 500 µm. (e) Schematic of optogenetic activation/inactivation of EC terminals during learning period of novel object recognition (NOR) test. (f,g) Discrimination index in NOR text by optogenetic activation (f) or inactivation (g) of subicular‐entorhinal circuits during learning period. (h) Schematic of optogenetic activation/inactivation of EC terminals during consolidation period of NOR text. (i,j) Discrimination index in NOR by optogenetic activation (i) or inactivation (j) of subicular–entorhinal circuits during consolidation period. (k) Schematic of optogenetic activation or inactivation of EC terminals during retrieval period of NOR test. (l,m) Discrimination index in NOR by optogenetic activation (l) or inactivation (m) of subicular–entorhinal circuits during retrieval period. Unpaired *t*‐test, **p < 0.01. (n) Schematic of optogenetic activation of ANT or MMB terminals during retrieval period of NOR test. (o,p) Discrimination index in NOR test by optogenetic activation of subicular–entorhinal circuits during retrieval period. Unpaired *t*‐test, *p < 0.05. The number of mice is indicated in the figure. Data are presented as mean± SEM.

To establish circuit‐specific causality, we optogenetically manipulated distinct subicular projections. We expressed AAV‐CaMKIIα‐ChR2‐eYFP or AAV‐CaMKIIα‐ArchT‐eYFP virus into the subiculum of WT mice and implanted optical cannula above the EC to enable real‐time photoactivation or inhibition of the subicular–entorhinal cortex circuits (Figure 2a,b). When light was delivered during learning or consolidation period, we found that neither activation nor inhibition of subicular projection terminals in the EC showed altered discrimination index in NOR. In contrast, when light was delivered during retrieval phase (Figure 2k), activation of this circuit significantly improved the discrimination index (Figure 2l, EYFP 31.50 ± 2.43% vs. ChR2 51.23 ± 4.96%, *p* = 0.0019), while photoinhibition of this circuit dampened discrimination index in NOR (Figure 2m, EYFP 35.73 ± 4.45% vs. ArchT 20.49 ± 2.54%, *p* = 0.0079). We validated the efficacy of optogenetic inhibition of the SUB–EC circuit by electrophysiological recordings, showing that pyramidal neurons in the EC exhibited sustaining reduced action potential (AP) firing following terminal inhibition (Figure S4). This manipulation was ineffective during learning or consolidation phases (Figure 2e–j), indicating temporal specificity.

Similarly, we aimed to illustrate whether and how the other two projecting pathways, the subicular–thalamic and –hypothalamic circuits were involved in the NOR. AAV‐CaMKIIα‐ChR2‐eYFP virus was injected into the subiculum of WT mice in like manner, and optical cannulas were implanted above the ANT or MMB (Figure 2c,d). We found that photoactivation of the subiculum–ANT and subiculum–MMB circuits during learning and consolidation phases did not alter the discrimination index (Figure S5a–f). Notably, activation of ANT‐projecting terminals during retrieval paradoxically disrupted novel object discrimination (Figure 2n,o, EYFP 37.31 ± 5.33% vs. ChR2 11.11 ± 9.36%, *p* = 0.0303), while MMB‐projecting terminals had no effect (Figure 2p). Moreover, we also tested whether inhibition of these two circuits would affect NOR behaviors, and found that neither optogenetic inhibition of subiculum–ANT nor –MMB circuits by ArchT changed NOR behaviors (Figure S5g–o). In addition, because the RSC is also an important downstream target of the subiculum and is closely associated with spatial navigation‐related behaviors [23], we examined the functional role of the SUB–RSC pathway during NOR. First, using cholera toxin b subunit (CTB)‐based retrograde tracing, we found that RSC‐projecting subpopulations and EC‐projecting subpopulations showed minimal overlap (Figure S6a–c). We then tested whether optogenetic inhibition of this pathway during different phases of the task affected NOR performance. The results showed that inhibition of the SUB–RSC circuit during the encoding, consolidation, or retrieval phases did not alter the discrimination index (Figure S6d–j). This is also consistent with the prevailing view that the RSC primarily processes “spatial” aspects of memory.

Moreover, we found that manipulation of subicular pyramidal neuron projecting terminals to the EC and ANT exclusively affects the retrieval of NOR, rather than OLR, since neither activation nor inhibition of subiculum–EC and –ANT during retrieval phase altered discrimination index in OLR (Figure S7), revealing a dedicated subiculum–EC circuit for NOR retrieval.

### 2.3 FN1 Defines a Molecular Substrate in the Subiculum for NOR Retrieval

**FIGURE 3:**
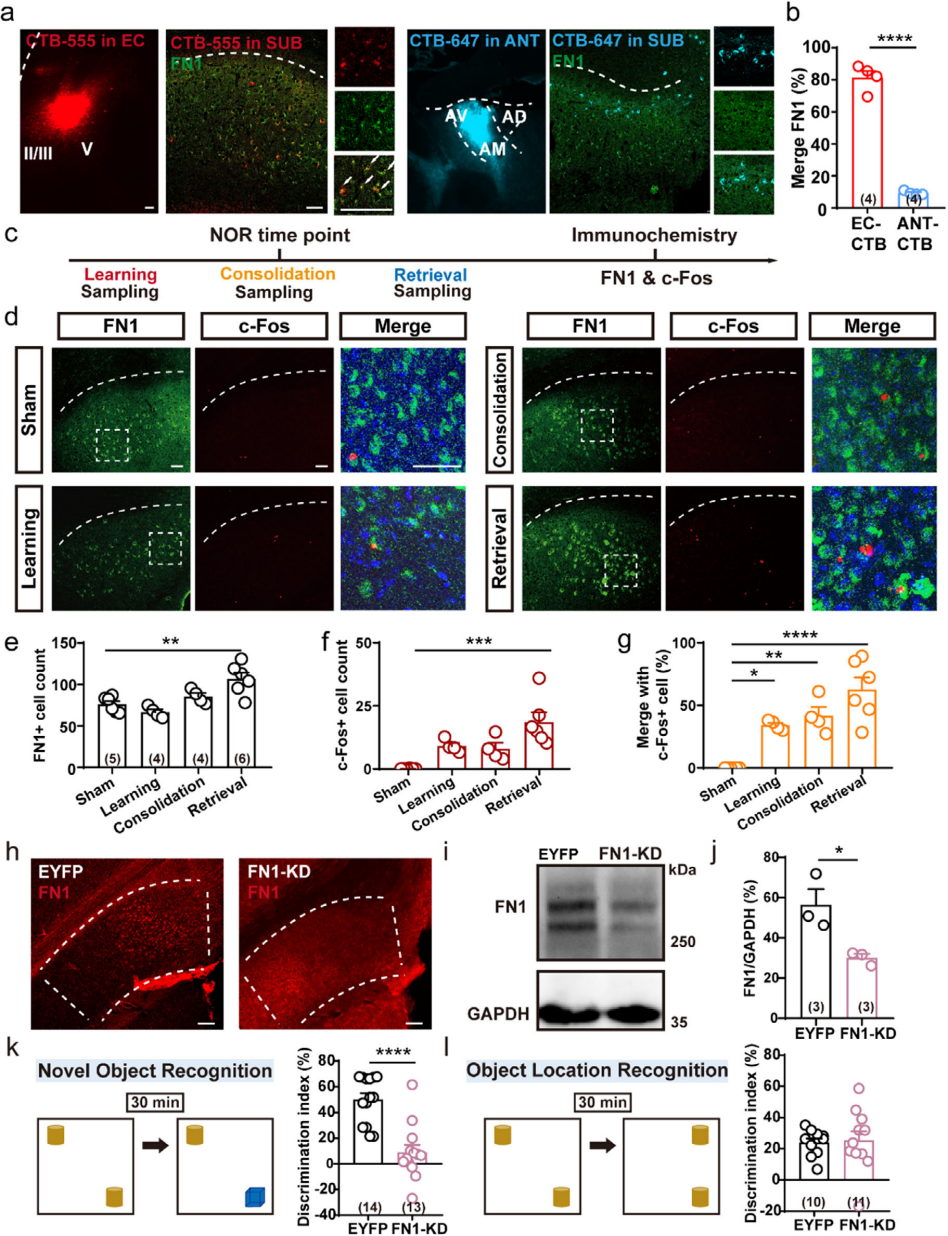
FN1 defines a functional molecular component in the subicular circuit for NOR retrieval. (a) Representative image of CTB‐555 injection in the entorhinal cortex (EC, red), CTB‐647 injection in the anterior nucleus of thalamus (ANT, cyan), CTB expression in the subiculum and colabeled with FN1 (green). Scale bar, 100 µm. (b) Quantification of CTB+ cell merged with FN1 marker in the subiculum. Unpaired *t*‐test, ****p < 0.0001. (c) Schematic of sampling for immunochemistry after different period of novel object recognition (NOR) test. (d) Representative images of FN1 expression (green) and colabeled with c‐Fos (red) after different period of NOR test. Scale bar, 50 µm. (e–g) Quantification of FN1+ (e), c‐Fos+ (f), and colabelled cells (g) after learning, consolidation, and retrieval periods. One‐way ANOVA with post hoc Tukey's multiple comparisons, *p < 0.05, **p < 0.01, ****p < 0.0001. (h) Representative images verifying subicular FN1 knockdown (KD). Scale bar, 500 µm. (i,j) Representative western blot (i) and quantification (j) of subicular FN1 protein before and after FN1‐KD. Unpaired *t* test, *p < 0.05. (k) Discrimination index in NOR test by FN1‐KD. Unpaired *t*‐test, ****p < 0.0001. (l) Discrimination index in object location recognition (OLR) by FN1‐KD. The number of mice is indicated in the figure. Data are presented as mean± SEM.

To dissect the subicular–entorhinal circuits in molecular level, we leveraged scRNA‐seq findings showing that FN1 is selectively expressed in the molecular layer of the subiculum [17], where pyramidal neurons are thought to project to the WC. To validate this finding, we injected CTB‐555 in the EC and found that FN1 expression was largely restricted to EC‐projecting subicular neurons, while ANT‐projecting neurons were almost all FN1‐negative (Figure 3a,b), suggesting FN1 as a molecular marker for subiculum–EC circuits.

Moreover, Fibronectin, a key component of the extracellular matrix, has been reported to regulate synaptic plasticity in the hippocampus [24]. To further investigate whether FN1 in the subiculum directly participates in the NOR, we first explored the expression of FN1 protein after the different phases by immunochemistry in different time points of behavioral phases (Figure 3c). The results showed that FN1 protein levels increased specifically during memory retrieval but not during learning or consolidation (Figure 3d,e, Sham 76.06 ± 3.78 vs. Retrieval 106.7 ± 7.57, *p* = 0.0030). This retrieval‐specific upregulation coincided with increased neuronal activity, as evidenced by enhanced c‐Fos expression and FN1/c‐Fos colocalization (Figure 3f,g, c‐Fos+ cell count, Sham 0.0550 ± 0.0550 vs. Retrieval 18.61 ± 3.806, *p* = 0.0002; FN1 adhesion to c‐Fos, Sham 0.00 ± 0.00 vs. Retrieval 62.69 ± 9.67, *p* < 0.0001). These results map the activity‐dependent activation pattern of subicular FN1, which specifies in the subicular–entorhinal circuits after retrieval process of NOR.

To test FN1's necessity in regulating NOR, we conditionally knocked down (KD) its expression by intrasubicular injection of pAAV‐CaMKIIα‐EGFP‐3xFLAG‐miR30 (FN1)‐WPRE virus, and then conducted the NOR test. The immunostaining and western blot results first confirmed that FN1 expression was significantly downregulated after FN1‐KD (Figure 3h–j). As expected, FN1‐KD severely impaired novel object discrimination while sparing novel location recognition (Figure 3k,l, discrimination index in NOR, EYFP 50.07 ± 4.90% vs. FN1‐KD 8.94 ± 5.89%, *p* < 0.0001; discrimination index in NLR, EYFP 23.90 ± 2.72% vs. FN1‐KD 25.26 ± 5.97%, *p* = 0.844). To verify that FN1 expression was activity‐dependently regulated in NOR, FN1 expression was then examined in mice with ArchT‐EYFP after either (i) administration of the AP‐1/c‐Fos inhibitor T5224 [25] into the subiculum 30 min after NOR retrieval, or (ii) optogenetic inhibition of subicular pyramidal neurons during the NOR retrieval phase (Figure S8a). We found that both manipulations markedly suppressed FN1 expression (Figure S8b–d), indicating that FN1 expression may be regulated by the immediate early gene protein c‐Fos, thereby participating in the NOR process.

Moreover, we also tested whether FN1‐KD in the subicular pyramidal neurons would affect other types of memory, and the results indicated that behavioral performance in spontaneous alternation of Y‐maze, latency to the platform of Morris water maze, and freezing duration of conditional fear memory test did not show significant difference (Figure S9), indicating FN1 in the subiculum specifically affect ‘object recognize’ memory. Additionally, FN1‐KD in the subicular pyramidal neurons neither affected emotional performance, i.e. anxiety and depression‐like behaviors (Figure S10).

### 2.4 Fibronectin1 Gates Subicular Neuronal Response in NOR Retrieval

**FIGURE 4:**
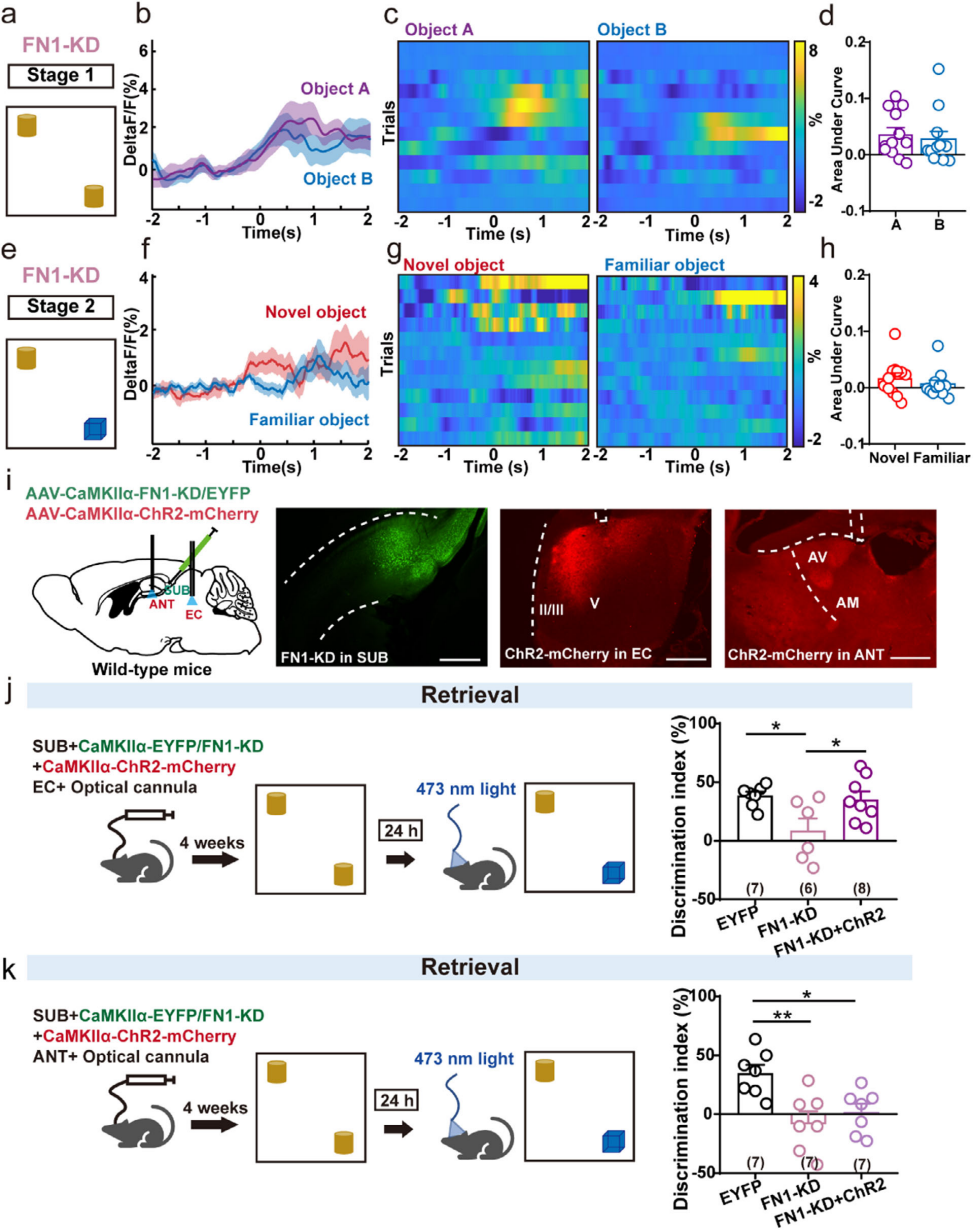
FN1 knockdown impairs NOR via modulating excitability of subicular‐entorhinal circuits. (a–c) Averaged values (b) and heatmap (c) of GCaMP6 (s) fluorescence of subicular pyramidal neurons after FN1‐KD during learning period of NOR test. (d) Area under curve (AUC) of both delta F/F lines in (b). (e–g) Averaged values (f) and heatmap (g) of GCaMP6 (s) fluorescence of subicular pyramidal neurons after FN1‐KD during retrieval period of NOR test. (h) AUC of both delta F/F lines in (f). *N* = 6 mice, each mouse included 2 trials. (i–k) Schematic of viral injection and representative images of viral expression and cannula placement in the entorhinal cortex (EC, j) and anterior nucleus of thalamus (ANT, k). Discrimination index in NOR test by subicular FN1‐KD combined with optogenetic activation of subicular–entorhinal or –thalamic circuits during retrieval period. One‐way ANOVA with post hoc Tukey's multiple comparisons, *p < 0.05,**p <0.01. The number of mice is indicated in the figure. Data are presented as mean ± SEM.

To understand how FN1 shapes the responses of subicular pyramidal neurons during NOR, we next performed calcium fiber photometry recordings in FN1‐KD mice. During the sample phase of the NOR task, subicular pyramidal neurons exhibited comparable calcium transients when mice explored the two identical objects, indicating intact encoding of object exploration (Figure 4a–d). However, during the test phase, calcium activity response was no longer distinguishable between exploration of the novel and familiar objects. This loss of differential activity suggests that, in FN1‐KD mice, subicular pyramidal neurons are no longer engaged in the retrieval of object‐related memory. (Figure 4e–h).

Further, we found that impairment in the NOR caused by FN1‐KD could be reversed by photoactivation of subicular–entorhinal circuits during retrieval phase (Figure 4I,j, EYFP 38.41 ± 3.43% vs. FN1‐KD 8.58 ± 10.66%, *p* = 0.0287; FN1‐KD vs. FN1‐KD‐ChR2 35.25 ± 6.77%, *p* = 0.0451). On the contrary, photoactivation of subicular–entorhinal circuits during the learning or consolidation phases did not rescue the impaired discrimination index induced by FN1‐KD (Figure S11a,b). We also examined whether photoactivation of subicular–thalamic circuits would affect altered NOR by FN1‐KD, and the results showed that this procedure neither increased nor decreased the recognitive behaviors in NOR tests (Figure 4k and Figure S11c,d). These results demonstrate that FN1 regulates retrieval of NOR via affecting excitability of subicular–entorhinal circuits.

### 2.5 Fibronectin1 Governs Intrinsic Excitability of Subicular Pyramidal Neurons by BK Channel Expression

**FIGURE 5:**
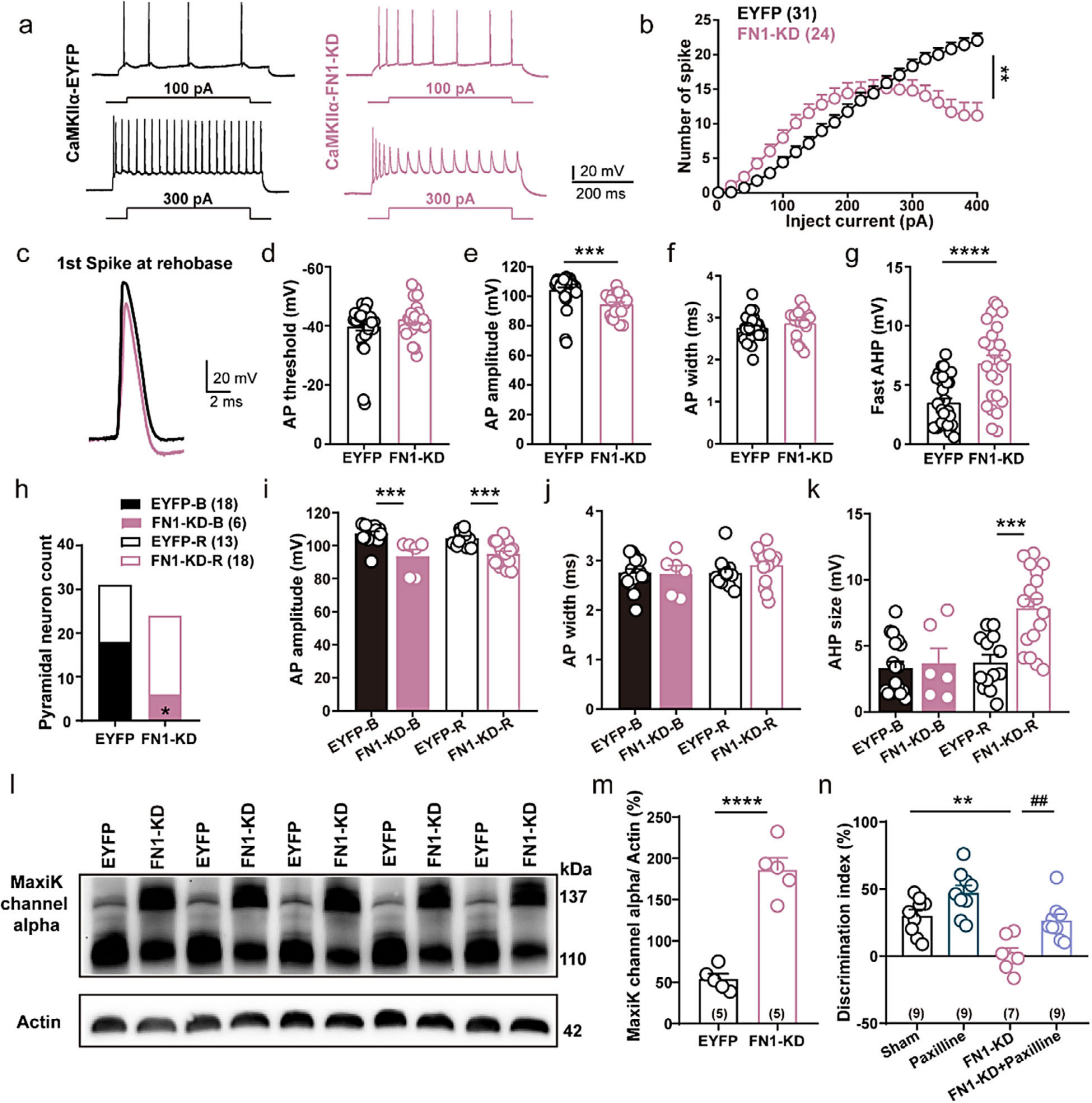
FN1 knockdown alters the intrinsic excitability of pyramidal neurons, likely in association with increased BK channel expression. (a) Representative action potential (AP) of subicular pyramidal neurons evoked by depolarization current (100 and 300 pA) by FN1 knockdown (KD). (b) Number of spike plot of subicular pyramidal neurons by FN1‐KD. Two‐way ANOVA test, **p < 0.01 (c) Representative first spike traces of subicular pyramidal neurons by FN1 KD. Note smaller amplitude and larger after hyperpolarization potential (AHP) size. (d–g) AP threshold (d), amplitude (e), width (f), and fast AHP size (g) of subicular pyramidal neurons by FN1‐KD. Unpaired *t*‐test, ***p < 0.001, ****p < 0.0001. (h) Number of bursting (B) and regular spiking (R) cells by FN1‐KD. Fisher's exact test, *p < 0.05. (i–k) AP amplitude (i), width (j), and fast AHP size (k) of subicular bursting and regular spiking cells by FN1‐KD. Two‐way ANOVA with post hoc Tukey's multiple comparisons test, ***p < 0.001. EYFP, *N* = 31 neurons from 6 mice. FN1‐KD, *N* = 24 neurons from 6 mice. (l,m) Representative western blot (l) and quantification (m) of subicular MaxiK channel alpha protein before and after FN1‐KD. Unpaired *t* test, ****p < 0.0001. (n) Discrimination index in NOR test by subicular FN1‐KD combined with intrasubicular injection of maxiK channel antagonist paxilline during retrieval period. One‐way ANOVA with post hoc Tukey's multiple comparisons test, **p < 0.01, ##*p* < 0.01. The number of mice is indicated in the figure. Data are presented as mean ± SEM.

To test how subicular FN1 affects neural excitability, we performed patch‐clamp experiments of subicular pyramidal neurons with EYFP or FN1‐KD. A series of hyperpolarizing–depolarizing current steps was injected into the patched neurons, and we found that although the rheobase of subicular pyramidal neurons was not significantly altered after FN1‐KD, they indeed exhibited a reduced number of action potentials in response to large depolarizing current injections (Figure 5a,b and Figure S12a,b). In terms of the first spike evoked by rheobase, we found that the action potential amplitude became smaller, and a notable larger fast after hyperpolarization potential (AHP) size were seen after FN1‐KD (Figure 5c–g, AP amplitude EYFP 104.3 ± 1.87 mV vs. FN1‐KD 94.65 ± 1.53 mV, *p* = 0.0003; fast AHP, EYFP 3.516 ± 0.36 mV vs. FN1‐KD 6.829 ± 0.69 mV, *p* < 0.0001). This type of afterhyperpolarization is generally considered to be mediated by large‐conductance Ca^2+^‐activated potassium (BK) currents [26]. While we did not observe a significant difference in the slow AHP (Figure S12d, EYFP 3.071 ± 0.21 mV vs. FN1‐KD 2.575 ± 0.28 mV, *p* = 0.161), which is typically associated with small‐conductance Ca^2+^‐activated potassium (SK) channels [27]. In addition, as subicular pyramidal neurons could be further divided into regular firing and bursting cells according to firing properties [28], we calculated the number of both neurons before and after FN1‐KD, and found that FN1‐KD specifically affected neuronal firing patterns by reducing the proportion of burst‐firing neurons (Figure 5h). Although both regular firing and bursting cells showed reduced action potential amplitude (Figure 5i), the increased fast AHP size was primarily observed in regular firing cells (Figure 5k). These results support the idea that FN1‐KD in the subicular pyramidal neurons leads to a general dampened intrinsic neuronal excitability especially in large depolarizing currents and one of mechanisms may be related to BK channel.

We then accordingly measured the expression of BK channel by western blot of subicular tissues after FN1‐KD and revealed a nearly fourfold increase in MaxiK channel alpha protein (the principal functional component of the BK channel in the central nervous system) expression following FN1‐KD (Figure 5l,m). To further investigate how FN1‐KD in the subiculum affects the protein‐level expression of the BK channel α‐subunit, we then assessed transcriptional changes in the two principal genes encoding BK channel subunits in the CNS using real time‐polymerase chain reaction (RT‐PCR). We found that FN1‐KD led to a significant upregulation of *KCNMA1* mRNA, whereas the expression of *KCNMB4* remained unchanged (Figure S13a,b). We next examined potential upstream regulators that may be directly influenced by alterations in extracellular ECM composition and have been reported to modulate *KCNMA1* transcription, including *Nrf2* and *CREB1* [29, 30]. In parallel, we also analyzed genes associated with BK channel α‐subunit protein degradation, such as *FBXO7* and *CRBN* [31]. RT‐PCR analysis revealed that among these candidates, *Nrf2* mRNA exhibited the most pronounced increase following FN1‐KD (Figure S13c–f). These findings suggest that the elevated expression of BK channels induced by FN1‐KD may be attributable to enhanced transcription of *KCNMA1*, rather than reduced protein degradation.

We further examined whether inhibition of BK channels could directly influence NOR performance in vivo. In the NOR model, mice with FN1‐KD showed decreased discrimination index in NOR, and this deficit was rescued by intrasubicular injection of selective BK channel blocker, paxiline [32] (Figure 5n, FN1‐KD 1.44 ± 4.79 % vs. FN1‐KD + paxiline 26.38 ± 4.80 %, *p* = 0.0094). These above results suggest that FN1‐KD in the subiculum show aberrant intrinsic excitability of pyramidal neurons, which may be due to the increased BK channel expression.

### 2.6 The CA1 Drives FN1‐Expressing Subicular–EC Circuits during NOR

**FIGURE 6:**
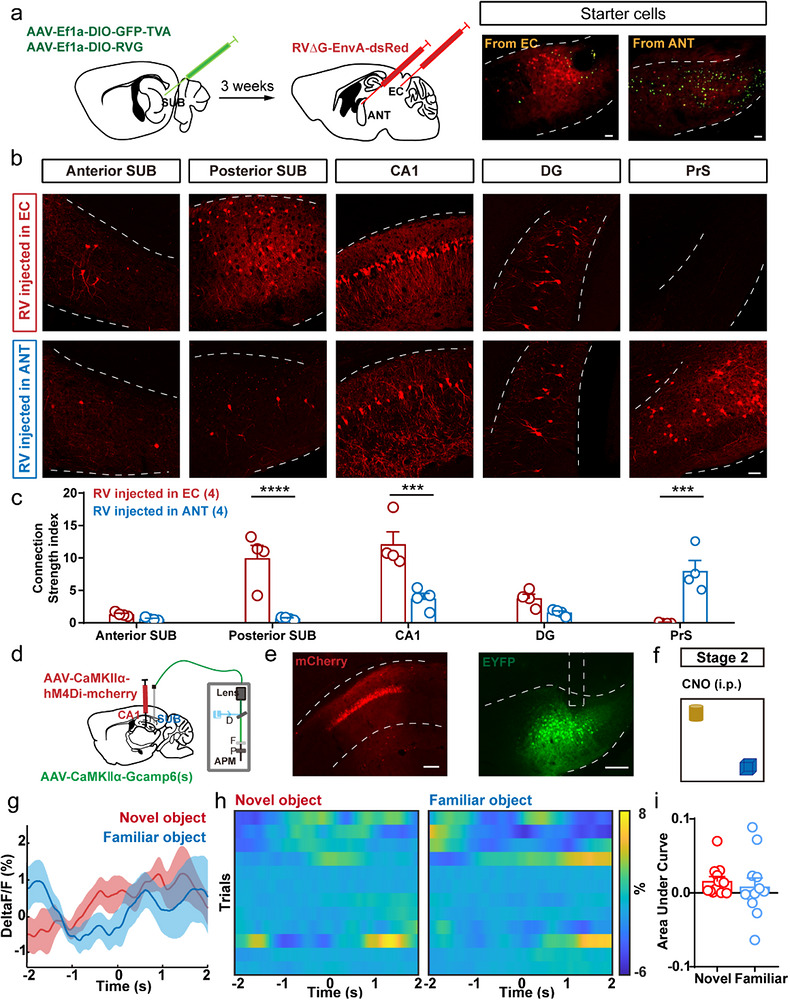
The CA1 innervates entorhinal‐projecting subicular pyramidal neurons and governs the retrieval of novel object recognition. (a) Left, scheme of TVA, RVG injection in the subiculum, and RV injection in the EC and ANT, respectively. Right, representative images of starter cells in the subiculum. (b) Representative images of RV+ neurons in the upstream areas of EC‐ and ANT‐projecting subicular glutamatergic neurons. Scale bar, 100 µm. (c) Connection strength index of RV+ neurons in (b). Two‐way ANOVA with post hoc Bonferroni's multiple comparisons test, ***p < 0.001, **** *p* < 0.0001. (d,e) Schematic of fiber photometry (d) and representative images of mCherry expression in the CA1 and GCaMP6(s) expression in the subiculum (e). (f,g) Averaged values (g) and heatmap (h) of GCaMP6(s) fluorescence of subicular pyramidal neurons during retrieval period after CNO injection. (i) Area under curve (AUC) of both delta F/F lines in (g). *N* = 6 mice, each mouse included 2 trials. The number of mice is indicated in the figure. Data are presented as mean ± SEM.

Finally, to systematically map brain‐wide afferents of FN1‐expressing subiculum–EC circuits involved in exploring the novel object, we employed circuit‐specific rabies virus (RV)‐mediated retrograde tracing. A mixture of helper viruses encoding tumor virus A (TVA) and rabies glycoprotein (RG) was first injected into the subiculum. Three weeks later, the glycoprotein‐deleted RVΔG was injected either into the EC or ANT, allowing for the specific labeling of presynaptic neurons targeting subicular pyramidal neurons projecting to these target regions. The distribution of starter cells was illustrated in Figure 6a. The results showed that subicular glutamatergic neurons projecting to the EC received robust innervation from the CA1 region and local microcircuits (Figure 6b,c, posterior SUB, RV injected in EC 9.96 ±1.98 vs. RV injected in ANT 0.66 ± 0.10, *p* < 0.0001; CA1, RV injected in EC 12.11 ± 1.91 vs. RV injected in ANT 3.75 ±0.80, *p* = 0.0003). In sharp contrast, those projecting to the ANT were predominantly innervated by the presubiculum (PrS), which notably did not project to EC‐projecting subicular neurons (Figure 6b,c, PrS, RV injected in EC 0.05 ± 0.05 vs. RV injected in ANT 7.99 ± 1.62, *p* = 0.0005). These findings indicate that the two subicular output pathways, subiculum–EC and subiculum–ANT circuits, are innervated by distinct upstream networks.

Given that CA1 glutamatergic neurons are critical for novel object recognition memory, we next assessed the functional relevance of CA1 input to the subiculum by chemogenetically inhibiting CA1 glutamatergic neurons while monitoring subicular activity during NOR (Figure 6d,e). Upon chemogenetical suppression of CA1 activity, subicular glutaminergic neurons failed to distinguish between novel and familiar objects based on calcium signals (Figure 6f–i). Together, these data suggest that FN1‐expressing EC‐projecting subicular neurons integrate the CA1 inputs, thereby coordinating hippocampal–cortical communication and playing a critical role in encoding NOR retrieval.

## 3 Discussion

As the principal output structure of the hippocampus, the subiculum establishes intricate interactions with cortical areas and plays an indispensable role in hippocampus‐related spatial navigation and memory. Notably, the subiculum has been identified as a critical hub within the hippocampal memory system, receiving preliminary information directly from the perirhinal and olfactory cortices and relaying it through the entorhinal–hippocampal “trisynaptic” circuits for further processing [33, 34, 35]. Moreover, supported by Deadwyler and Hampson report, the subiculum encodes and maintains novel information in a highly accurate and specific manner for 10–15 s before the hippocampus [36], suggesting that the subiculum may possess distinct memory‐related functions that operate independently of the canonical hippocampal memory system. Our findings extend these views by demonstrating that the subiculum plays a specific role in NOR retrieval through direct modulation of downstream EC circuits.

Although the structure and function of the SUB/CA1–EC circuitry and its involvement in cognitive functions are well established in the field [37, 38], we found that selectively inhibiting the subiculum–EC circuits, but not projections to the ANT, MMB, or RSC, specifically impairs NOR while preserving other memory modalities, that is, this circuit plays a dominant role specifically in cognitive (object‐related) memory rather than spatial memory. Intriguingly, activation of the parallel subiculum–ANT circuits instead impairs NOR, highlighting the functional heterogeneity of subicular circuits. Indeed, the subiculum is characterized by distinct topological organization, with specific neuronal populations projecting to different downstream regions. For example, the distal subiculum (i.e., the region far away from CA1) preferentially innervates the posterior cingulate cortex, anterior hypothalamus, and medial EC, whereas the proximal subiculum (adjacent to CA1) primarily projects to the nucleus accumbens, perirhinal cortex, and lateral EC [18, 35]. This topographically organized projection pattern suggests that the subiculum may allocate distinct information‐processing functions to discrete downstream regions. Supporting this notion, previous studies have suggested that subicular pyramidal neuron projections to the MMB mediate stress hormone responses following fear memory retrieval, while the distal subiculum, but not the proximal region, is preferentially involved in spatial memory encoding [13, 18]. Conversely, subicular pyramidal neurons that project back to the CA1 (predominantly located in proximal) mediate object location learning processes [15]. Our study delineates the critical function of the subiculum–EC circuits in NOR retrieval while demonstrating that this circuit is not strongly associated with OLR memory. This functional specificity aligns with recent evidence indicating that the subiculum does not exhibit a familiarity bias in spatial location tasks [39]. Furthermore, we demonstrate that the EC‐ and ANT‐projecting subicular pyramidal neurons can be further distinguished based on their upstream neural inputs. Specifically, the CA1 region may functionally interact with EC‐projecting subicular neurons in the regulation of NOR retrieval. In fact, sensory signaling input is crucial for the imitation of memory perception and retrieval [40, 41]. As we did not find any RV cell in the visual or auditory cortex, whether the neural afferents from these regions indirectly influence the EC‐projecting subicular pyramidal neurons by innervating the CA1 needs further studies. Collectively, these findings provide new insights into the specialized functional architecture of subicular circuits and their distinct contributions to NOR.

The subiculum exhibits remarkable heterogeneity not only in its circuit organization but also in its molecular composition. Our findings reveal that pyramidal neurons projecting to the EC strongly express FN1, whereas FN1 expression is virtually absent in pyramidal neurons projecting to the ANT. Using CTB‐assisted retrograde tracing, we further find that the subpopulations of subicular neurons projecting to the RSC and to the EC showed minimal overlap, suggesting that these two projection pathways are unlikely to represent structurally identical “shared” neuronal populations, although both may contain FN1‐positive neurons, as reported in the recent study [42]. Consistent with this anatomical distinction, our behavioral data indicate that the SUB–RSC pathway does not mediate NOR, which aligns with the prevailing view that the RSC primarily processes the spatial components of memory [23, 43]. The specific functional role of FN1‐positive subicular neurons projecting to the RSC therefore remains to be elucidated in future studies. Notably, FN1 expression in the central nervous system appears to be exclusive to subicular pyramidal neurons, with no detectable presence in CA1 or other adjacent regions. Recent transcriptomic analyses further corroborate its predominant expression in the distal molecular layer [42, 44]. Our results indicate that FN1 selectively governs the NOR retrieval by regulating the excitability of pyramidal neurons in the EC‐projecting molecular layer.

Over the past few decades, the functions of FN1 in peripheral tissues have been extensively characterized, particularly its role as an adhesion protein in maintaining vascular integrity and driving tumor progression [45, 46]. In contrast, its contributions to neural function remain unexplored. Previous studies have suggested that neuronal FN1 is implicated in processes such as cell migration and axonal regeneration [47]. Our results first found that regulation of FN1 could selectively influence NOR retrieval by showing that FN1 knockdown in subicular pyramidal neurons selectively increases the fast AHP but not the slow AHP of regular firing cells. This effect is mediated by the upregulation of BK channel expression, leading to reduced neuronal excitability at larger injecting current and impaired NOR, likely via disrupted subiculum–EC circuits function. We would like to emphasize that the effect of FN1‐KD on subicular pyramidal neurons is not a simple reduction in neuronal excitability. At low current injections, FN1‐KD instead shows a tendency to enhance excitability, although this effect does not reach statistical significance. In contrast, at higher current injections, FN1‐KD markedly suppresses neuronal excitability. Such a characteristic is likely associated with BK channel's Ca^2^^+^‐dependent gating. Specifically, insufficient Ca^2^^+^ influx under low current leads to minimal channel opening, whereas robust Ca^2^^+^ influx upon high excitability triggers strong channel activation, which in turn induces rapid repolarization and posthyperpolarization, thereby inhibiting subsequent neuronal firing [26]. Previous studies have reported that the spontaneous firing rate of subicular pyramidal neurons in rodents is approximately 1–5 Hz under resting conditions, whereas during certain cognitive behaviors this firing rate can increase substantially [48, 49]. We therefore speculate that during cognitive tasks, subicular FN1+ pyramidal neurons are strongly engaged and operate in a high‐excitability network. Under such conditions, the suppressive effect of FN1‐KD on network integration becomes more prominent, ultimately leading to impaired performance in the NOR task. Indeed, BK channels play an important role in regulating neuronal excitability by facilitating rapid action potential repolarization [50]. We find that FN1 knockdown, through upregulation of BK channels, significantly reduces the proportion of bursting neurons, a finding consistent with previous studies in cortical neurons and cerebellar Purkinje cells [51, 52]. Given that bursting activity is essential for efficient information transmission in the subiculum [53], this reduction likely contributes to the observed deficits in NOR retrieval. Additionally, while BK channels have been reported to regulate resting membrane potential in some certain neurons [54], we do not observe this effect in our study, perhaps because FN1‐KD also affect the expression of other membrane channels, such as voltage‐gated sodium channels, since the spike amplitude value as well sharply decreases. Nonetheless, our results indicate that FN1 deletion profoundly alters BK channel expression, thereby affecting the excitability of subicular pyramidal neurons. Unfortunately, at present we have not identified an effective approach to upregulate FN1 expression or enhance its function, likely due to its relatively large molecular weight. Nevertheless, one potential mechanism by which FN1 overexpression enhances neuronal excitability is through the suppression of BK channel activity. Accordingly, by directly increasing the excitability of subicular neurons that project to the EC via upregulation of FN1 expression, we are able to achieve a functional enhancement in NOR performance.

As FN1 has crucial function in cell adhesion and migration [55], it is plausible that alterations in FN1 levels may influence overall cellular morphology and polarity, thereby affecting the membrane distribution and functional properties of potassium channels. Although a substantial body of literature highlights a close interplay between the ECM, synaptic plasticity, and neuronal excitability, there is currently no well‐defined experimental framework to directly dissect their functional interconnections in this specific context. Our findings provide preliminary evidence that reduced FN1 levels may directly enhance the transcription and translation of *KCNMA1*, which encodes BK channel α‐subunit, potentially mediated by the upregulation of key upstream transcription factors such as *Nrf2* and *CREB1*. An interesting hypothesis is that sensory input signals preferentially activate EC‐projecting subicular pyramidal neurons, this neural activity may lead to the induction of immediate early gene protein such as c‐Fos, drive the transcription of ECM components (like FN1) and upregulate their protein expression [56]. Following FN1‐KD, ECM remodeling [24], or alterations in integrin‐dependent signaling pathways involving focal adhesion kinases, Rho GTPases, and related molecules may occur [57], ultimately leading to changes in the transcriptional activity of key regulators such as *Nrf2*, supported by ECM‐integrin signaling has been reported to modulate *Nrf2* pathways, particularly in cardiovascular and stress‐related conditions [58]. Notably, direct transcriptional regulation of the *KCNMA1* gene by *Nrf2* or *CREB1* has also been reported in other cell types, supporting the plausibility of this pathway. In addition, *Nrf2* may also modulate BK channel α protein expression through nontranscriptional mechanisms, an area that warrants further investigation [30, 59]. Taken together, these pathways may converge to influence BK channel function and postsynaptic membrane excitability, thereby contributing to the observed impairment in NOR performance following FN1‐KD [60, 61]. Importantly, we cannot exclude the possibility that additional ECM components or regulatory mechanisms within the subiculum also contribute to the modulation of overall neuronal excitability, thereby influencing novelty recognition. However, the precise mechanisms by which FN1 regulates BK channel expression and modulates the excitability of subicular neurons remain unclear, representing an important limitation of the present study. Together, beyond serving as a molecular marker for the subiculum–EC circuits, our study also demonstrates that FN1 itself plays a previously unknown role in regulating NOR via the intrinsic excitability of subicular pyramidal neurons, establishing a link between FN1 and BK channel. Indeed, BK channels are involved in multiple physiological and pathological processes, for instance, epilepsy and Parkinson's disease. In epilepsy, the BK channel β4 subunit has been identified as a key regulator of intrinsic firing properties in DG neurons, playing a protective role against seizure activity [62]. Pharmacological BK channel modulators are currently being explored in preclinical models for the treatment of neurological conditions, including cerebellar ataxia and movement disorders, with the goal of ameliorating disease progression [63, 64]. Our study suggests that excessive BK channel activity in the subiculum impairs recognition memory, an effect that can be reversed by the selective BK channel antagonist paxilline, highlighting a potential therapeutic target for cognitive dysfunction.

In summary, we identify a specialized molecular mechanism whereby FN1‐expressing subicular neurons encode novel object preference and selectively govern retrieval of NOR via affecting excitability of EC‐projecting circuits, this effect is likely mediated through the modulation of BK channels. These findings provide new insights into the molecular and circuit‐level mechanisms underlying the subiculum's function in NOR and may offer novel targets for therapeutic intervention in cognitive disorders.

## 4 Materials and Methods

### 4.1 Animals

This study used male *C57BL/6J* (WT) mice (purchased from SLAC ANIMAL, China) and *CaMKIIα‐Cre* (stock number: 005359) transgenic mice. The *CaMKIIα‐Cre* mice were genotyped by the guideline from Jackson laboratory. All animals were group‐housed in cages (5–6 per cage, temperature 20–26°C, relative humidity 40%–70%) under a 12 h light‐dark cycle (light intensity 15–20 lux, light hours 8:00–20:00), with free access to food and water. All experimental procedures were approved by the ethical committee of Zhejiang Chinese Medical University (No. 17150) and in complete compliance with the National Institutes of Health Guide for the Care and Use of Laboratory Animals.

### 4.2 Viral Conducts

For calcium photometry, 100 nL AAV2/9‐CaMKIIα‐GCaMP6s (2.05 × 10^12^ vg/mL, OBio Technology, China) or AAV2/8‐DIO‐Axon‐GCaMP6s (5.6 × 10^12^ vg/mL, Taitool Bioscience, China) virus was injected into the subiculum of WT or *CaMKIIα‐Cre* mice, respectively. For chemogenetics, 100 nL AAV2/8‐CaMKIIα‐hM3D(Gq)‐mCherry (1.4 × 10^13^ vg/mL, OBio Technology, China), AAV2/8‐CaMKIIα‐hM4D(Gi)‐mCherry (1.4 × 10^13^ vg/mL, OBio Technology, China), or AAV2/8‐CaMKIIα‐mCherry (1.4 × 10^13^ vg/mL, OBio Technology, China) virus was injected into the subiculum of WT mice. For optogenetics, 100 nL AAV2/8‐CaMKIIα‐hChR2 (H134R)‐EYFP (1.7 × 10^12^ vg/mL, OBio Technology, China), AAV2/8‐CaMKIIα‐ArchT‐EGFP (1.61 × 10^12^ vg/mL, OBio Technology, China), or AAV2/8‐CaMKIIα‐EYFP (1.7 × 10^12^ vg/mL, OBio Technology, China) virus was injected into the subiculum of WT mice. For retrograde tracing, 100 nL Cholera Toxin Subunit B conjugated to Alexa Fluor 555/647 fluorophore (CTB‐555 or CTB‐647, 1.0 mg/mL, Thermo Fisher Scientific, USA) was injected into the EC or ANT of WT mice. For selectively knockdown of FN1, the following target sequence: *CCGTTTTCATCCAACAAGA* (Gene ID: NM_001276408.1), and 100 nL pAAV‐CaMKIIα‐EGFP‐3xFLAG‐miR30(FN1)‐WPRE (1.45 × 10^13^ vg/mL, OBio Technology, China), or pAAV‐CaMKIIα‐3xFLAG‐miR30(FN1)‐WPRE (1.45 × 10^13^ vg/mL, OBio Technology, China) was used and injected into the subiculum of WT mice. For monosynaptic retrograde tracing, 60 nL AAV2/9‐hEF1α‐DIO‐TVA‐RVG‐eGFP (viral titers: 1.0 × 10^13^ particles/mL, Taitool Bioscience, China) were injected into the EC or ANT, and 90 nL RV‐EnvA‐∆G‐mCherry (viral titers: 1.0 × 10^13^ particles/mL, Taitool Bioscience, China) were injected into the subiculum of *CaMKIIα‐Cre* mice.

All virus were stored at −80°C until use.

### 4.3 Stereotactic Injections and Surgeries

Mice were anesthetized with ∼2% isoflurane and mounted in a stereotaxic apparatus (68043, RWD life Science, China) for viral injection and stereotactic surgery. During this period, body temperature of the mice was kept warm via a heating pad. For AAV injection, a glass micropipette attached to a 1 µL syringe was fixed in the injection pump (Pump 11 Elite, Harvard, USA). Injections were target into the subiculum (AP −3.4 mm; L −2.0 mm; DV −1.8 mm). The injection speed was kept at 50 nL/min and the injection volume was 150 nL. For CTB injection, injections were target into the EC (AP −4.8 mm; L −4.0 mm; DV −3.5 mm), ANT (AP −0.6 mm; L −0.7 mm; DV −3.7 mm), and RSC (AP −2.8 mm; L −0.2 mm; DV −0.8 mm). The injection speed was kept at 20 nL/min and the injection volume was 80 nL. After each injection, the needle of the syringe was left for 10 min before withdrawal. The skin was then closed using surgical clips.

3–4 weeks after injection, optical fiber cannula (0.2 mm diameter, 0.37 NA, 6 or 8 mm terminal length, Inper, China) was then inserted into the subiculum (AP −3.4 mm; L −2.0 mm; DV −1.7 mm), EC (AP −4.8 mm; L −4.0 mm; DV −3.3 mm), ANT (AP −0.6 mm; L −0.7 mm; DV −3.5 mm), MMB (AP −2.8 mm; L −0.2 mm; DV −4.9 mm), or RSC (AP −2.8 mm; L −0.2 mm; DV −0.8 mm) for light delivery. For intrasubiculum drug administration, 0.41 mm diameter cannula (62003, RWD Life Science, China) was inserted into the subiculum. Three screws were placed over the skull separately to fix the dental cement. After surgeries, mice were allowed to recover for 1 week to conduct behavioral tests. All mice were checked for viral expression and cannula location after behavioral tests. Only mice with limited viral expression and correct cannula location were included in further analysis.

### 4.4 Behavioral Tests

All behavioral experiments were conducted between 9:00 a.m. and 17:00 p.m. in mature male mice at ages of 3–5 months old. The specific procedure follows our previous studies [65, 66]. Briefly, the mice were handled by the same experimenter for 10 min per day for five days before experiments to reduce the stress reactions caused by fear or anxiety. The experimental apparatus was cleaned with 10% ethanol after each animal to remove odors and any residues. All mice were video‐recorded their behaviors.

#### 4.4.1 Novel Object and Object Location Recognition

The mice were habituated to the clear experimental apparatus (45 × 45 × 45 cm) for 1 h to freely explore in the day before sample day. The NOR or OLR test comprised 2 stages. In stage 1 (sample stage), two identical objects were put in the opposite position of the apparatus, then the mice were put in the same apparatus in same direction and allowed to explore. The recording session last for 10 min and then the mice were taken out. 30 min or 24 h later, we performed stage 2 (test stage). During this stage, mice were returned to the apparatus for the 5 min test. In NOR test, mice were allowed to explore one copy of the object in the same location as in the sample stage and the other novel object (same size, but novel shape and color) in the same location. In OLR test, mice were allowed to explore one copy of the object in the same location as in the sample stage and one copy of the object in a novel location. The exploring behavior was defined as the distance between the nose of mice and the object was less than 1 cm. The duration when mice climbing or staying on the object was excluded in calculation. The data were further analyzed by ANYMAZE software. The discrimination index equaled to (Time exploring novel object/object with novel location − Time exploring familiar object)/ (Time exploring novel object/object with novel location + Time exploring familiar object) × 100% in stage 2.

#### 4.4.2 Spontaneous Alternation Behavior in Y Maze

The Y‐shape maze comprised three arms (A, B, and C, 10 × 40 × 16 cm) at a 120° angle from each other. The experiment began by placing the mice in the center of the maze. Mice were allowed to freely explore in 8 min. Spontaneous alternation behavior was defined as the mice continually exploring three different arms, i.e., ABC, ACB, BCA…, but not ABB. Percentage of spontaneous alternation was equaled to (Number of spontaneous alternations / (Total arm entries − 2)) × 100%.

#### 4.4.3 Morris Water Maze

The water maze apparatus was made up by a dark circular pool with 120 cm diameter and 30 cm depth. The water temperature was maintained at 21–23°C. The circular pool was divided into four quadrants by two imaginary vertical lines through the center. In the middle of one of the quadrants, a platform with a diameter of 10 cm was placed at a distance of about 2 cm from the surface, and was hidden by powdered starch. The experiment lasted for 6 days. On the first day, the mice were placed and allowed to swim freely for 60 s to acclimatize. From the second day to the fifth day, the mice were put into the water from four different quadrants, i.e., NW, SW, NE, and SE quadrants, with their backs facing the wall for 60 s. If the mice found the platform successfully in 60 s, they were allowed to stay on the platform for 5 s and then taken out; if the mice failed to find the platform, then the time was recorded as 60 s and the mice were guided to the platform and then taken out of the water after 10 s. Each quadrant was determined in a pseudo‐randomized manner, with a 20 min interval between each water entry. The sixth day was the test day, in which the hidden platform was removed and the mice were placed into the water at the entry point of the opposite quadrant of the platform. The time spent in each quadrant and the number of times the mice passed through the platform location within 60 s were recorded and analyzed.

#### 4.4.4 Conditional Fear Memory

Conditional fear memory test was performed in a box with metal plate (Environment A), which enabled electrical foot shock of the mice. On the day before training, the mice were placed into the device for 5 min. To test context and cue memory separately, the mice were subjected to a total of 3 day tests. On the first day, the mice were placed in the device (Environment A) and allowed to explore freely for 2 min, and then were subjected to three noise‐shock paired training sessions, in which a noise (5000 Hz, 85 dB) was emitted for 30 s at the third, fourth, and fifth minute of the experiment, respectively, and a shock was applied for 2 s at the end of the noise each time (0.5 mA, 2 s). For context memory, the mice were placed in Environment A and allowed to explore freely for 5 min without noise. For cue memory, mice were placed in an opaque acrylic cylinder with black‐white stripes (Environment B, 15 cm in diameter and 15.5 cm in height) and allowed to explore freely for 5 min. Mice were stimulated with noise (5000 Hz, 85 dB) for the last 3 min. Freezing time was analyzed automatically by Freezeframe software and checked manually.

#### 4.4.5 Acoustic Startle in Prepulse Inhibition (PPI)

The PPI test was conducted using SR‐LAB startle chambers. Each mouse was placed individually in the test chamber, and the session commenced with a 5 min acclimation period during which a continuous background white noise of 70 dB was maintained. This was followed by a block of five 20 ms startle pulses at 120 dB to measure baseline startle responses. Subsequently, six blocks of trials were conducted to evaluate PPI. Each trial block included eight different trial types, presented in a pseudo‐randomized order across blocks: (1) startle tone only, (2) no stimulus, (3) pulse tones at 74, 78, or 82 dB, and (4) pulse tones paired with a 120 dB startle tone. The session concluded with an additional block of five 20 ms startle pulses at 120 dB. For each trial, the maximum amplitude of the startle response was recorded. The average response for each trial type across the six blocks was calculated and used for statistical analysis. Percent PPI was determined using the following formula: [1 − (averaged startle response to prepulse before startle stimulus/averaged response to startle stimulus)] × 100%.

#### 4.4.6 Tail Suspension Test

Mice were allowed to habituate in the test room for 30 min and then gently suspended by the tail for 6 min. Immobility time of this period was recorded.

#### 4.4.7 Forced Swim Test

Mice were allowed to habituate in the test room for 30 min and then placed in a clear glass cylinder filled with water for 6 min. The temperature of the water was maintained at 21–22°C. Immobility time of this period was recorded.

#### 4.4.8 Light‐Dark Box Test

Light‐dark box test was conducted in an apparatus consisted of a light chamber (∼600 lux, 15 × 20 × 25 cm) and a dark chamber (∼5 lux, 30 × 20 × 25 cm). A small hole was opened to enable mice freely moving between two chambers. Mice were placed into the center of the light box and then allowed to explore for 5 min. The time spent in the light box was measured.

#### 4.4.9 Open Field Test

Open field test was conducted in a clear experimental apparatus (45 × 45 × 45 cm). Mice were placed into the center of the apparatus and then allowed to explore for 30 min. The data were further analyzed by ANYMAZE software. Distance moved was analyzed per 5 min and in total. Time and distance moved in the center zone (%) were also calculated.

#### 4.4.10 Three‐Chamber Test

Three‐chamber test was conducted in an apparatus consisted of a box (60 × 40 × 20 cm) divided into three chambers. The whole test contained three phases: Phase 1‐Habituation: the left and right chambers both contained empty wire cages (E). The mouse was placed in the central chamber and allowed to freely explore for 10 min. Phase 2‐Sociability: an age‐ and gender‐ matched stranger mouse (S1) was placed into a wire cage. The test mouse was placed in the center chamber and allowed to freely explore for 10 min. The total time spent in each chamber and close interaction time with the cages were measured. Phase 3‐Social novelty recognition: a new stranger mouse (S2) was placed into the other empty cage. The test mouse was placed in the center chamber and allowed to explore for 10 min. The total time spent in each chamber and close interaction time with the cages were measured. The data were further analyzed by ANYMAZE software. Preference index (S1 − E) was measured as the ratio of (S1 − E) to (S1 + E) and preference index (S2 − S1) was measured as the ratio of (S2 – S1) to (S2 + S1).

### 4.5 Calcium Fiber Photometry

Calcium fiber photometry was conducted in mice expressing GCaMP6 (s) during NOR according to our previous study [67]. The fiber photometry system (Nanjing Thinkertech) used a 488 nm diode laser reflected by a dichroic mirror (MD498, Thorlabs), and then coupled into an objective lens. The laser intensity between the fiber tip and the animal ranged from 0.01 to 0.03 mW to avoid bleaching. Mice were placed into the apparatus 30 min before recording to adapt the environment. Then they were recorded baseline activity for 3 min. Next, two identical objects were put in the opposite position of the apparatus, the mice were recorded for 10 min in the sample stage. 30 min later, one of the objects was replaced by the other one of the same size, different color and shape, and the calcium signaling was recorded for another 5 min. Experimental data were further analyzed by MATLAB software. Δ*F*/*F* was calculated by (*F* − *F*_0_) /*F*_0_, in which *F*_0_ represented the average baseline activity. The Δ*F*/*F* values of each mouse were presented in the form of Δ*F*/*F*‐time plot and heatmap. In these plots, the zero point on the *x*‐axis represents the timing when the mouse's nose tip entered the object zone (<1 cm). Area under curve was calculated as the value below the Δ*F*/*F* line.

### 4.6 Light Stimulation

For light delivery in optogenetics, the 200 µm diameter fiber was connected to the laser device. Mice those expressed ChR2 or eYFP received 473 nm blue light (Laser intensity: 3–5 mW, 20 Hz, 10 ms pulse, 10 s on–off), those expressed ArchT or eYFP received 589 nm yellow light (Laser intensity: 3–5 mW, 20 Hz, DC, 10 s on–off). For manipulating the learning phase, light stimulation was delivered throughout the entire learning session. For the consolidation phase, light stimulation was applied 6 h after the learning session for a duration of 10 min. For the retrieval phase, light stimulation was delivered throughout the entire retrieval session.

### 4.7 In Vivo Pharmacology

During in vivo pharmacology, mice were intraperitoneal injected with CNO (1.0 mg/kg, ab141704, Abcam, USA).

For manipulation of the learning phase, CNO was administered 15 min before the first testing session. For manipulation of the consolidation phase, CNO was administered 6 h after completion of the first testing session. For manipulation of the retrieval phase, CNO was administered 15 min before the second testing session, while no CNO was given during the first phase.

For local delivery of T5224 (500 um, 300 nL, HY‐12270, MedChemExpress, USA) and paxiline (50 µm, 300 nL, HY‐N6778, MedChemExpress, USA), drugs were administrated through a cannula guided into the subiculum in free moving mice.

### 4.8 In Vitro Electrophysiology

Mice were decapitated and then brains were rapidly dissected and placed in ice‐cold oxygenated solution containing the following substances (in mm): 110 choline chloride, 2.5 KCl, 1.3 NaH_2_PO_4_, 25 NaHCO_3_, 0.5 CaCl_2_, 7 MgCl_2_, 20 D‐glucose, 1.3 ascorbic acid, and 0.6 Na‐pyruvate. 300 µm coronal slices containing the hippocampus and para‐hippocampal regions were cut by a vibratome (VT1000, Leica, Germany) and incubated at 34.7°C for 0.5 h and then maintained at 25°C for further experiments.

Patch‐clamp recordings were performed as in our pervious study [68, 69]. Patch pipettes (6–8 MΩ) were filled with an intracellular solution containing (in mm): 140 K gluconate, 5 NaCl, 10 HEPES, 0.2 EGTA, 2 Mg‐ATP (pH 7.20 adjusted with KOH, 290–300 mOsm). Signals were amplified and recorded by a HEKA EPC10 amplifier (HEKA Instruments, Germany). Cells in the subiculum of control and FN1‐KD mice were patch‐clamped. For plotting of spike‐current functions, a gradient of depolarizing currents (0 to 400 pA, 20 pA each step, 500 ms) were injected. For measurement of rheobase and AP properties, a gradient of depolarizing currents (−50 pA to 200 pA, 5 pA each step, 500 ms) were injected. Rheobase was defined as the minimum depolarizing current that elicits the first AP spike. AP threshold, amplitude, width and AHP size were calculated in the first AP spike. Bursting cell was defined as one in which action potential emitted by 100 pA current was clusters, while regular firing cell was single. For validation of ArchT‐based optogenetic inhibition, we performed patch‐clamp recordings on entorhinal cortex neurons. Baseline AP firing was first recorded for 20 s, followed by continuous light stimulation for 20 s, and finally for another 20 s after light offset.

### 4.9 Western Blot

The mice were deeply anesthetized with sodium pentobarbital, and their brains were extracted and perfused with 0.9% saline. The subiculum was isolated using a mouse brain mold. Tissue samples were homogenized in RIPA buffer (20 mmol/L Tris‐HCl, pH 7.5; 150 mmol/L NaCl; 1 mmol/L EDTA; 1% Triton X‐100; 0.5% sodium deoxycholate; 1 mmol/L PMSF; 10 µg/mL aprotinin). Protein concentrations were quantified, and 100 µg of protein from each sample was separated by sodium dodecyl sulfate‐polyacrylamide gel electrophoresis (SDS‐PAGE), then transferred onto a nitrocellulose membrane. The membrane was blocked with 5% skim milk in phosphate‐buffered saline (PBS) for 1 h at room temperature and then incubated overnight at 4°C with the primary antibody at the appropriate dilution. The primary antibodies used were as follows: rabbit anti‐Fibronectin (1: 1,000; Ab2413, abcam, USA), mouse anti‐Maxi potassium channel alpha (1: 1,000; Ab192759, abcam, USA), rabbit anti‐β‐actin (1: 1,000; AC026, Abclonal, China), mouse anti‐GAPDH (1: 1,000; AC002, Abclonal, China). The following day, the membrane was allowed to equilibrate to room temperature for 30 min, then washed 3 times with TPBS for 15 min each. The membrane was then incubated with the appropriate secondary antibody dilution for 2 h at room temperature, followed by three 15 min washes with TPBS. Protein bands were visualized using chemiluminescence with an Odyssey imaging system (LI‐COR Biosciences) and the ECL Western Blotting Detection Kit (61809, Sungky Bio Tech). Densitometric analysis of the images was performed using Image‐Pro Plus software (USA), and relative protein levels were normalized to internal reference proteins.

### 4.10 Real Time‐PCR

Subiculum tissues of EYFP and FN1‐KD groups were removed and homogenized using an automated tissue homogenizer, and the supernatant was collected by centrifugation. After that, the total RNA was extracted according to the manufacturer's instructions of the RNAeasy Animal RNA Isolation Kit. RNA concentration and purity were assessed using a microplate reader, and samples with an OD260/OD280 ratio between 1.8 and 2.0 were used for subsequent experiments. RNA was reverse transcribed into cDNA at 37°C for 15 min, 85°C for 5 s, and held at 4°C. Amplification was accomplished with 7500 Real‐Time PCR System and a reaction mixture made up of the Taq SYBR Green PCR Premix. Relative transcription levels of mRNA genes were calculated using the2‐ΔΔCT method. Primer sequences used included (5ʹ– 3ʹ): GAPDH‐F: ACTCACGGCAAATTCAACGG;

GAPDH‐R: CTTCAGCTTTCCGGCCACTTA;

KCNMA1‐F: CGCCTCTTCATGGTCTTCTTCAT;

KCNMA1‐R: TTTCTTCCACTAACCGCGCTATA;

KCNMB4‐F: GTACACGGAAGCCGAAGACA;

KCNMB4‐R: CAGGTGAAGGTGCACTCGAA;

Nrf2‐F: TGGCAGAGACATTCCCATTTGTA;

Nrf2‐R: CTTGCTCCATGTCCTGCTCTATG;

CREB1‐F: GGAAGAGAGAGGTCCGTCTAATG;

CREB1‐R: GCACTGCCACTCTGTTCTCTAAA;

CRBN‐F: CAGTCTGCCAACCTCACATACAT;

CRBN‐R: AATCAGGATCATCAGCACCTCAG;

Fbxo7‐F: CATTTGATGCCTGGACTGATGAC;

Fbxo7‐R: CCTGCCCATCCTCTGTTTCATTA.

### 4.11 Immunohistochemistry and Cell Counting

For c‐Fos and FN1 immunostaining, the Sham control group consisted of mice that had only been habituated to the environment. The remaining three groups were sampled at 1.5 and 7.5 h after the first stage, and at 1.5 h after the second stage, respectively. During tissue collection, the mice were deeply anesthetized with sodium pentobarbital, and their brains were extracted and perfused with 0.9% saline and 4% PFA. The brains were fixed in 4% PFA overnight at 4°C, then transferred to a 30% sucrose solution for 48 h for dehydration. Coronal sections (40 µm) were cut using a freezing microtome (NX50, Thermo Scientific, USA) and stored at 4°C.

RV cell counting was performed as in our pervious study [69], 60 µm slices were selected from bregma location approximately +2.46 mm to −4.96 mm with 60 µm interval. Slices containing each corresponding regions were all included in calculation by ImageJ 8.0 (Fuji, Japan) software. The connection strength index was defined as presynaptic cell number in certain brain region/starter cell number.

For immunostaining, sections were rewarmed for 30 min and incubated for 2 h in a blocking solution (PBS + 3% bovine serum albumin (BSA) + 5% normal donkey serum + 0.3% Triton X‐100), followed by overnight incubation with primary antibodies at 4°C. The primary antibodies used in the present study were: rabbit anti‐CaMKII (1:800; Ab134041, abcam, USA), rabbit anti‐Fibronectin (1: 200; Ab2413, abcam, USA), rabbit anti‐c‐Fos (1:500; Ab208942, abcam, USA). After three 10 min PBS washes, sections were incubated with the appropriate secondary antibodies for 2 h at room temperature, followed by three additional washes. Finally, slices were mounted on glass slides with DAPI (Vectashield Mounting Media, Vector Labs). Images were captured using a confocal microscope (SP8, Leica, Germany).

For c‐Fos+ and FN1+ cell counting, three consecutive coronal sections from the same subicular region were selected for each experimental group. FN1 and c‐Fos colocalization is defined as the proportion of neurons that are simultaneously positive for c‐Fos, FN1, and DAPI signals, relative to the total population of neurons positive for both c‐Fos and DAPI. Cytoplasmic localization of c‐Fos under this criterion. The average value of three sections was calculated. Viral expression levels were quantified using ImageJ software (USA) and are presented as integrated density values.

### 4.12 Statistics and Reproducibility

Experiments and data analyses were conducted by investigators who were blinded to experimental groups. Data are presented as mean ± standard error of the mean (SEM). Only mice with correct viral expression and placement of cannulas were included in the analysis. All experiments were independently repeated at least twice, yielding similar results. Statistical analyses were performed using Prism (version 9.0, GraphPad software, USA). For comparisons between two groups, Unpaired *t*‐test or paired *t*‐test was applied, while comparisons among multiple groups were analyzed using one‐way ANOVA, Fridman test or two‐way ANOVA followed by appropriate post‐hoc tests. All tests were two‐sided, and a *p* value < 0.05 was considered statistically significant. All statistical details and values were provided in a supplementary table attached to the manuscript.

## Author Contributions

Z.C. and Y.W. conceived and supervised the study; F.F., J.Y.S., and C.L. X. designed the experiments; F.F., J.Y. S., J.X., Y.X.X., S.Y.Y., X.K.F., and M.H.L. performed the experiments and analyzed the data; W.J.L.L., Z.S.L., Y.W., L.C., L.Y., and L.Y.X. help with results interpretation and discussion; F.F. and J.Y.S. wrote the manuscript; Z.C., C.L.X., and Y.W. revised the manuscript.

## Conflicts of Interest

The authors declare no conflicts of interest.

## Supporting information




**Supporting File**: advs75264‐sup‐0001‐SuppMat.docx.

## Data Availability

The data that support the findings of this study are available in the supplementary material of this article.
