# Supplementary material for: Fibronectin1‐Expressing Subicular Circuits Selectively Govern the Retrieval of Novel Object Recognition
Source: Adv Sci (Weinh). 2026 Apr 15;13(39):e16399. doi: 10.1002/advs.202516399 (PMC13334995; doi:10.1002/advs.202516399)
Supplement: Supplementary file 1 — Supporting File: advs75264‐sup‐0001‐SuppMat.docx. [file ADVS-13-e16399-s001.docx]

*Supplementary information for*

**Fibronectin1-expressing Subicular Circuits Selectively Govern The Retrieval of Novel Object Recognition**

Fan Fei ^1, 2#^, Jiaying Shi ^2#^, Jing Xi ^2#^, Yixiang Xu ^1#^, Shuye Ying ^1^, Xukun Fan ^1^, Wangjialu Lu ^2^, Zhisheng Li ^2^, Menghan Li ^1^, Yu Wang ^1^, Li Cheng ^1^, Lin Yang ^1^, Lingyu Xu ^1^, Zhong Chen ^1, 2*^, Cenglin Xu ^1*^ and Yi Wang ^1, 2*^

^1^ Zhejiang Collaborative Innovation Center for the Brain Diseases with Integrative Medicine, Zhejiang Key Laboratory of Neuropsychopharmacology, School of Pharmaceutical Sciences, & First Affiliated Hospital, Zhejiang Chinese Medical University, Hangzhou, 310053, China

^2^ Institute of Pharmacology and Toxicology, College of Pharmaceutical Sciences, School of Medicine, Zhejiang University, Hangzhou, 310058, China

# These authors contributed equally to this work.

**Corresponding author:**

Prof. Yi Wang, Ph. D.

E-mail: [wang-yi@zju.edu.cn](mailto:wang-yi@zju.edu.cn)

Prof. Cenglin Xu, Ph.D.

E-mail: [xucenglin5zz@zju.edu.cn](mailto:xucenglin5zz@zju.edu.cn)

Prof. Zhong Chen, Ph. D.

E-mail: [chenzhong@zju.edu.cn](mailto:chenzhong@zju.edu.cn)


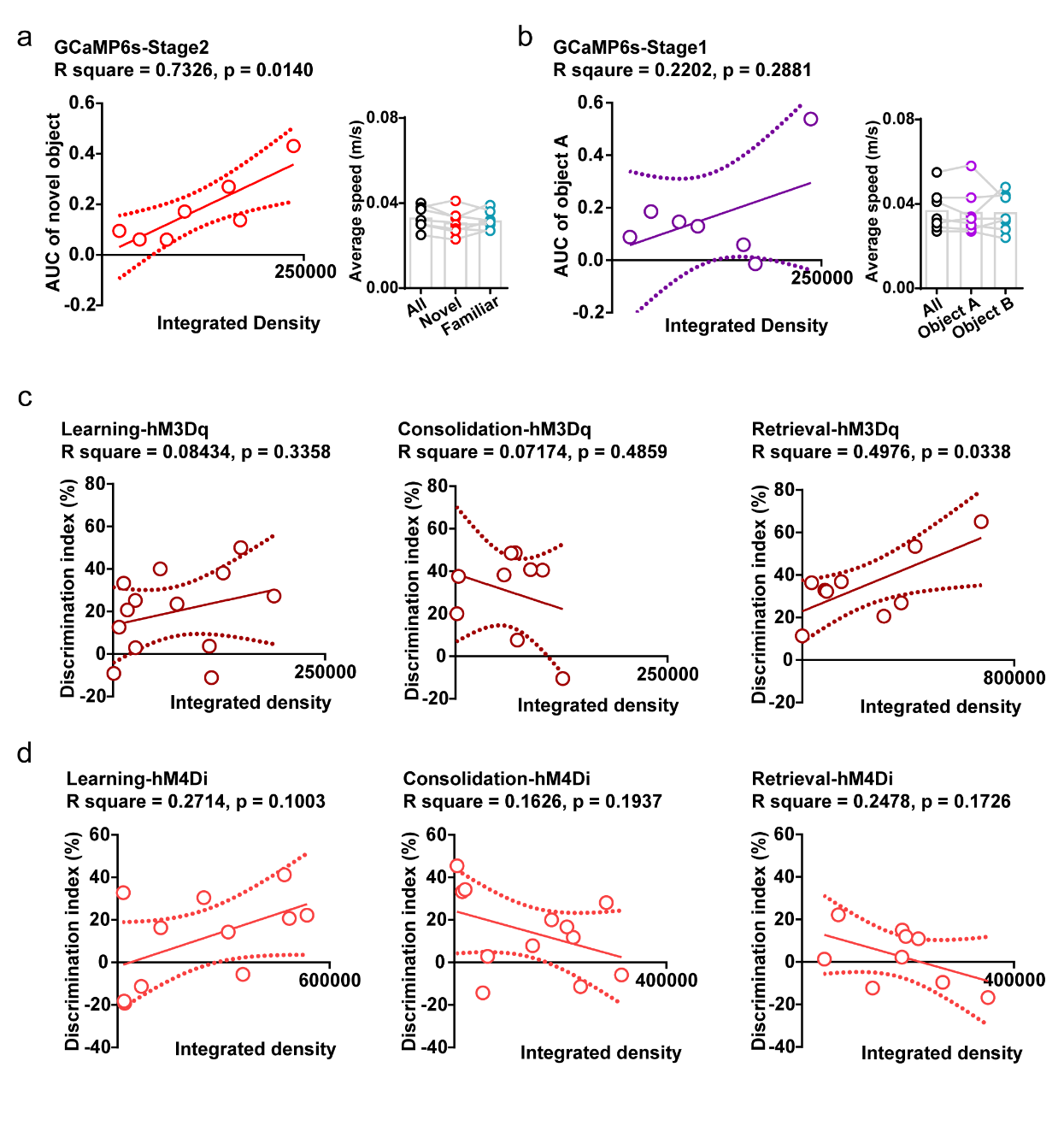


**Supplementary Figure 1. Correlationship between** **viral expression and changes in calcium signaling or NOR behaviors. a** Left, linear relationship between GCaMP expression and area under curve (AUC) of calcium signaling during stage 2 of each mouse. Right, average speed of mouse during the whole test (All), exploring novel object (Novel) and exploring familiar object (Familiar). **b** Left, linear relationship between GCaMP expression and AUC of calcium signaling during stage 1 of each mouse. Right, average speed of mouse during the whole test (All), exploring A object (Object A) and exploring B object (Object B). **c** Linear relationship between hM3Dq-mCherry expression and changes in discrimination index of NOR behaviors. **d** Linear relationship between hM4Di-mCherry expression and changes in discrimination index of NOR behaviors. Data are presented as separated dots.


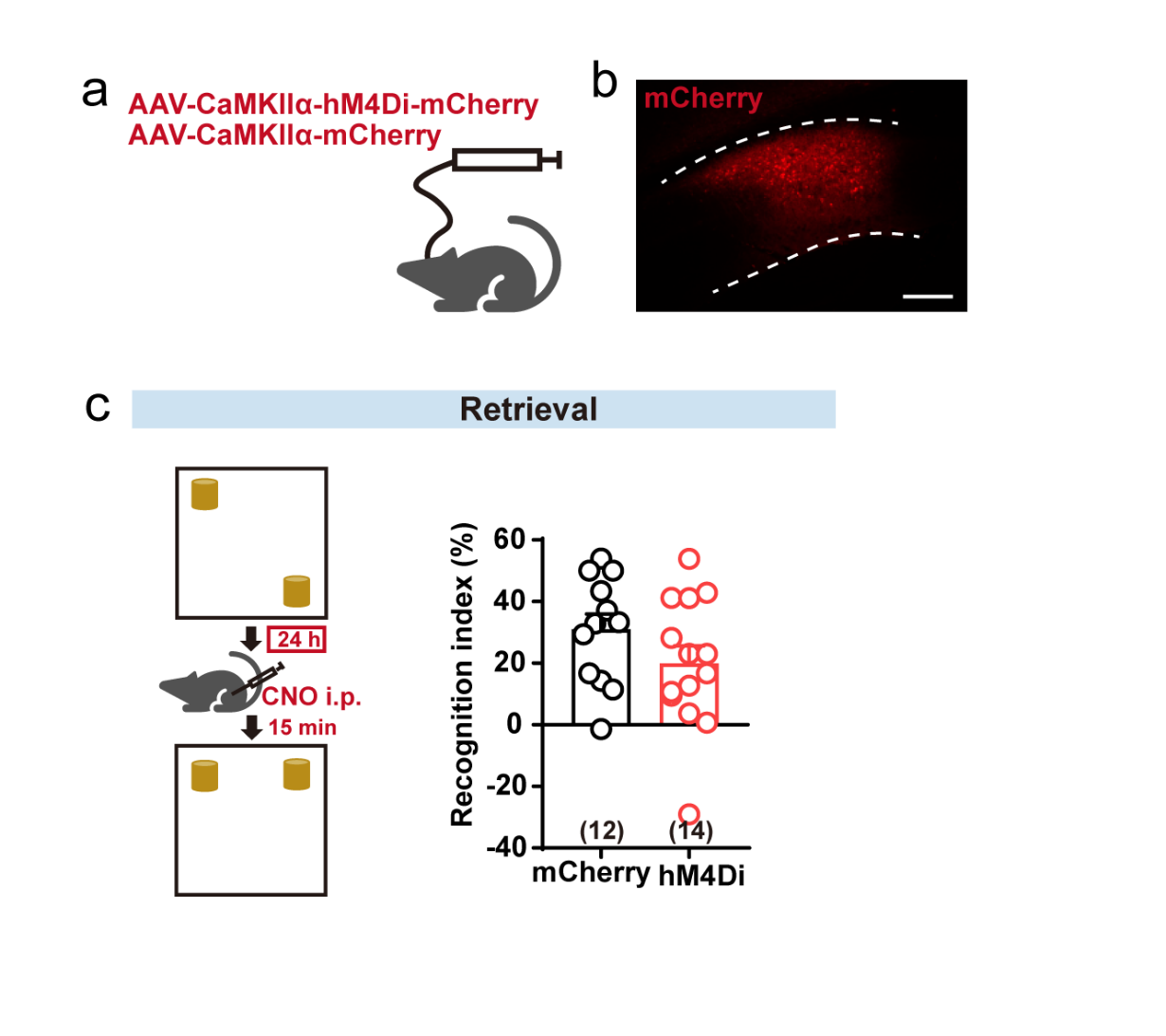


**Supplementary Figure 2. Effect of chemogenetic inhibition of subicular pyramidal neurons on the object location recognition retrieval.** **a** Schematic of viral injection. **b** Representative image of mCherry expression in the subiculum. Scale bar, 200 μm. **c** Discrimination ratio in the object location recognition (OLR) test by chemogenetic inhibition of subicular pyramidal neurons during retrieval period. The number of mice is indicated in the figure. Data are presented as mean± S.E.M.


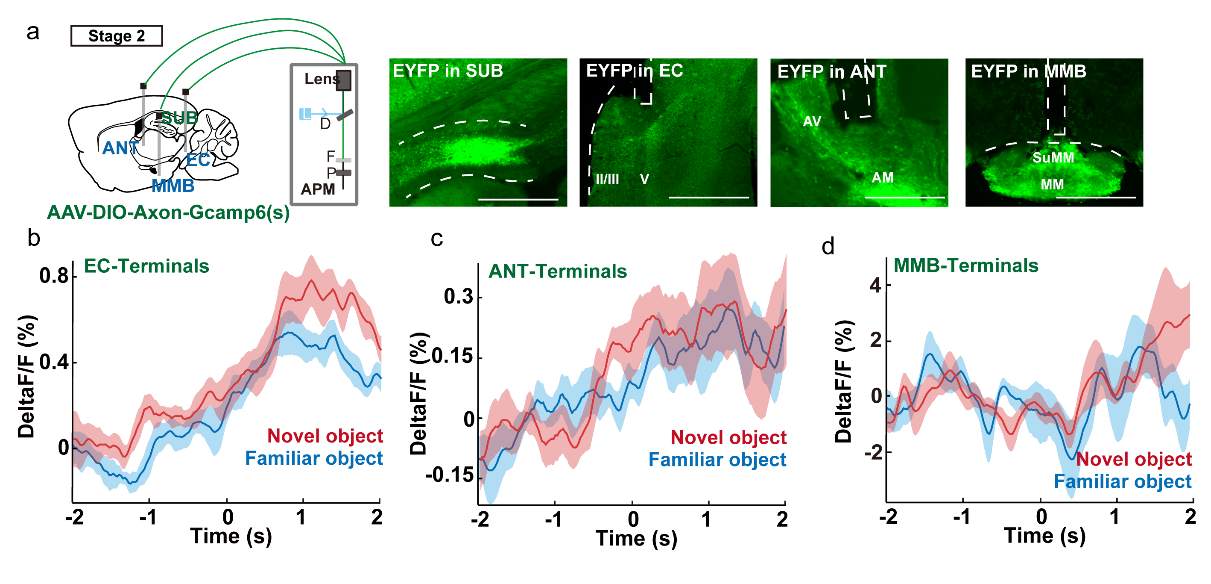


**Supplementary Figure 3. The subicular-entorhinal circuit manifests novel object preference during the NOR retrival. a** Schematic of fiber photometry and representative image of GCaMP6(s) expression in the subicular terminals. Scale bar, 1 mm. **b** Averaged values of GCaMP6 (s) fluorescence of entorhinal terminals during NOR retrieval. N = 5 mice, each mouse included 2 trials. **c** Averaged values of GCaMP6 (s) fluorescence of thalamic terminals during NOR retrieval. N = 6 mice, each mouse included 2 trials. **d** Averaged values of GCaMP6 (s) fluorescence of hypothalamic terminals during NOR stage 2. N = 5 mice, each mouse included 2 trials. Data are presented as mean± S.E.M.


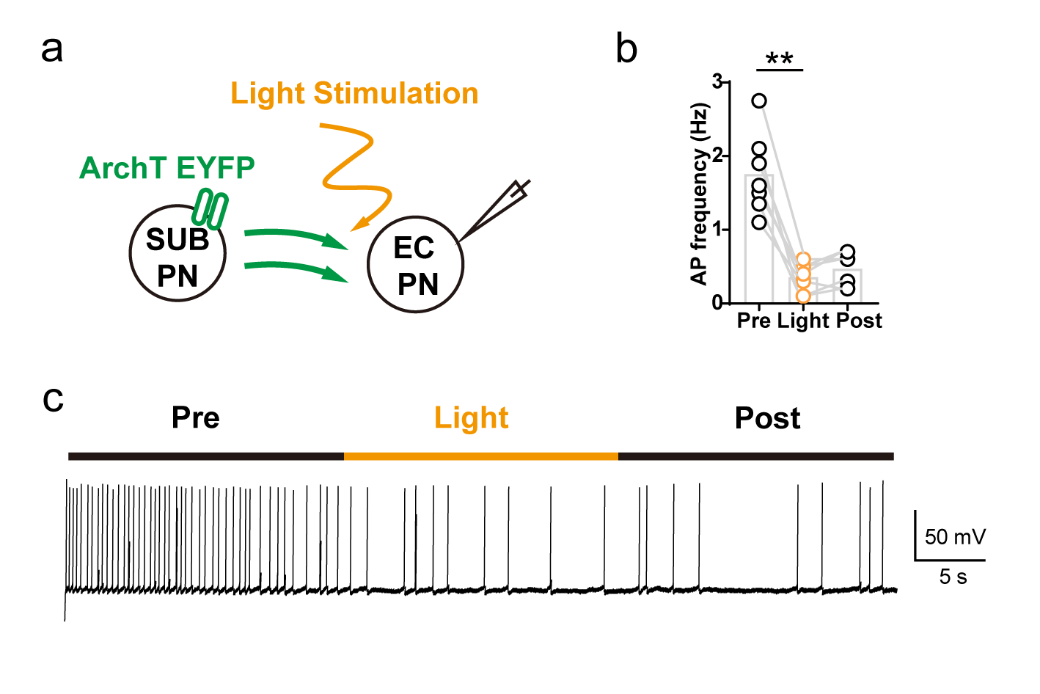


**Supplementary Figure 4. Functional validation of optogenetic inhibition of subicular-entorhinal terminals. a** Scheme of 589 nm light stimulation in the subicular pyramidal neurons expressed with ArchT and patch recording in the EC pyramidal neurons. **b** Action potential (AP) frequency before (Pre), during (Light) and after (Post) stimulation. N = 7 neurons from 2 mice. Friedman with *post hoc* Dunn's multiple comparisons test, ***p*<0.01. **c** Representative AP firing traces from a recorded EC neuron before and during light stimulation. Data are presented as mean with separated dots.


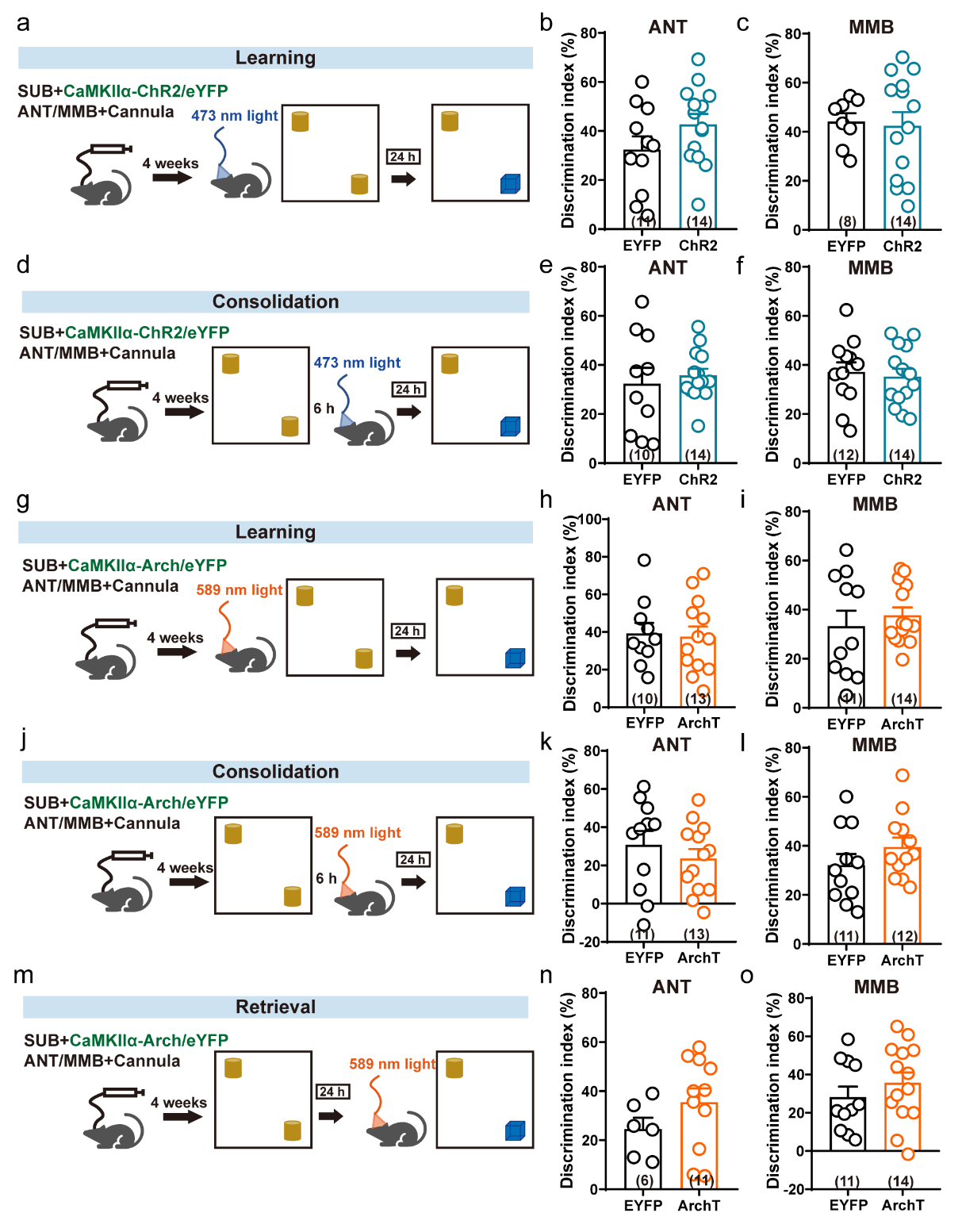


**Supplementary Figure 5. Manipulation of subicular-thalamic and subicular-hypothalamic circuits during different stages of NOR.** **a** Schematic of optogenetic activation during learning period of novel object recognition (NOR). **b** and **c** Discrimination index in NOR by opto-activation of subicular-thalamic (**b**) or -hypothalamic (**c**) circuits during learning. **d** Schematic of optogenetic activation during consolidation period of NOR. **e** and **f** Discrimination index in NOR by opto-activation of subicular-thalamic (**e**) or -hypothalamic (**f**) circuits during consolidation. **g** Schematic of optogenetic inactivation during learning period of NOR. **h** and **i** Discrimination index in NOR by opto-inactivation of subicular-thalamic (**h**) or -hypothalamic (**i**) circuits during learning. **j** Schematic of optogenetic inactivation during consolidation period of NOR. **k** and **l** Discrimination index in NOR by opto-inactivation of subicular-thalamic (**k**) or -hypothalamic (**l**) circuits during consolidation. **m** Schematic of optogenetic inactivation during retrieval period of NOR. **n** and **o** Discrimination index in NOR by opto-inactivation of subicular-thalamic (**n**) or -hypothalamic (**o**) circuits during retrieval. The number of mice is indicated in the figure. Data are presented as mean± S.E.M.

**
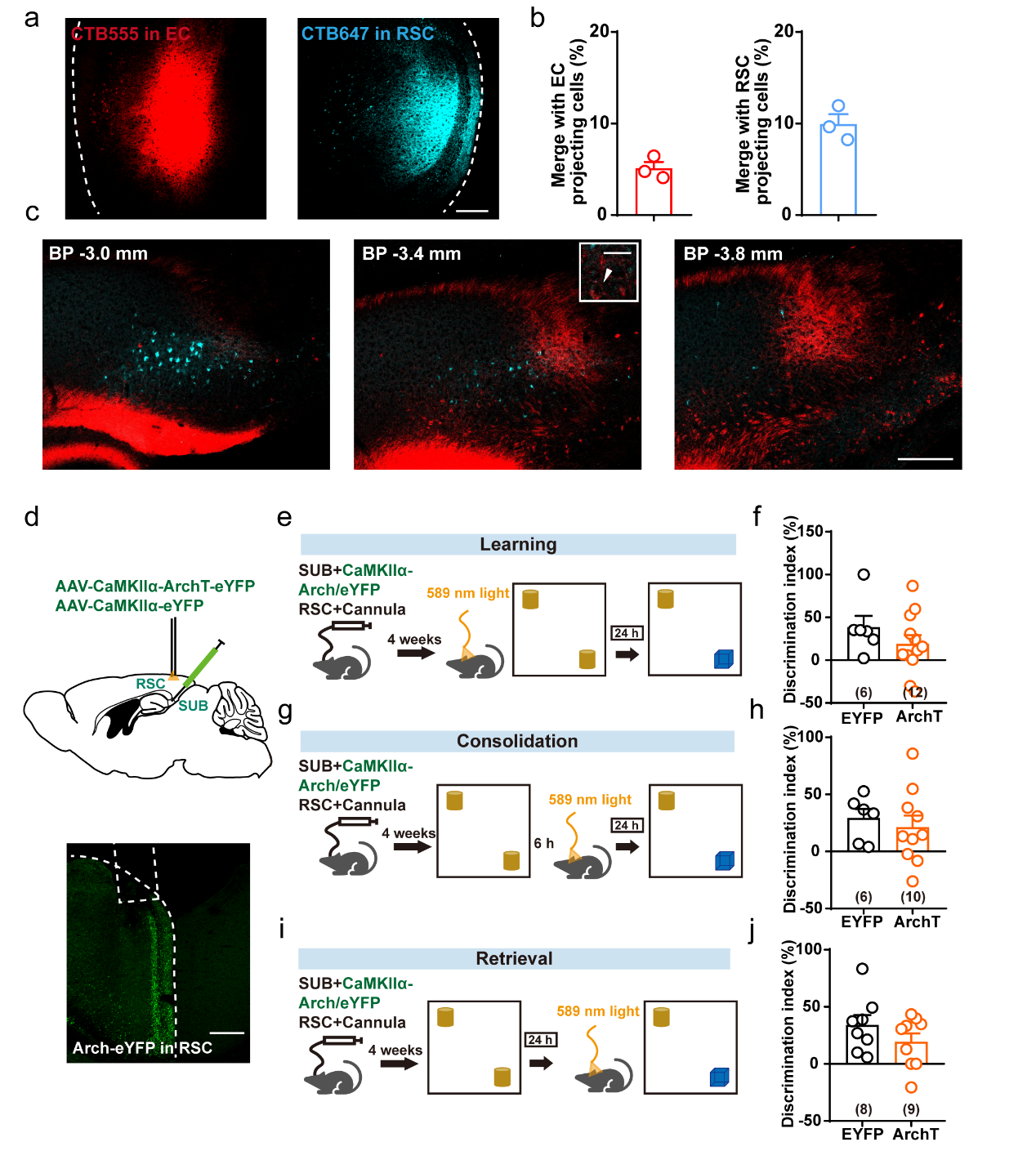
**

**Supplementary Figure 6. Manipulation of subicular-retrosplenial cortex during different stages of NOR. a** Representative image of CTB-555 injection in the entorhinal cortex (EC, red), CTB-647 injection in the retrosplenial cortex (RSC, cyan). Scale bar, 200 μm. **b** Quantification of double labelled cells merge with EC-projecting cells and RSC-projecting cells. N = 3 mice. **c** Representative image of CTB-555 and CTB-647 expression in the subiculum. Scale bar, 200 μm. **d** Schematic of viral injection in different groups and representative images of viral expression and cannula placement in the RSC. Scale bar, 200 μm. **e-j** Schematic and discrimination index in NOR by optogenetic inactivation of subicular-retrosplenial circuits during learning (**e** and **f**), consolidation (**g** and **h**) and retrieval period (**i** and **j**). The number of mice is indicated in the figure. Data are presented as mean± S.E.M.


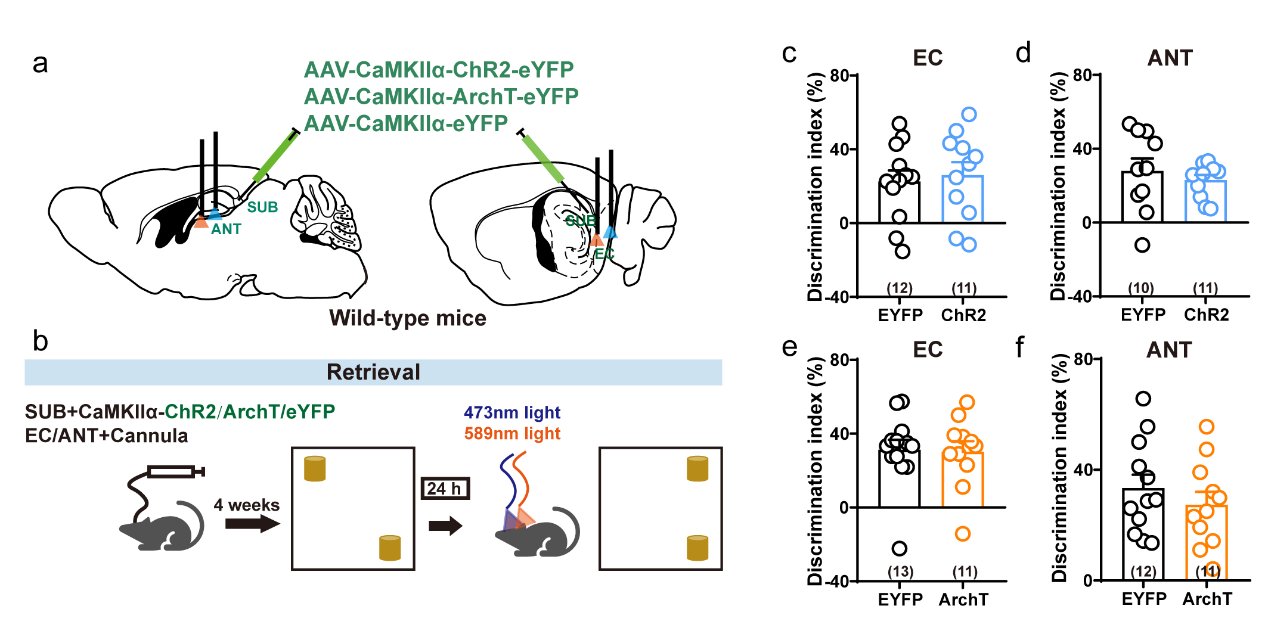


**Supplementary Figure 7. Subicular-entorhinal and -thalamic circuits do not involve in the OLR retrieval. a** Schematic of AAV injection and cannula placement. **b** Schematic of optogenetic manipulation during retrieval period of object location recognition (OLR) test. **c** and **d** Discrimination index in OLR test by opto-inactivation of subicular-entorhinal (**c**) or -thalamic (**d**) circuits during retrieval. **e** and **f** Discrimination index in OLR test by opto-inactivation of subicular-entorhinal (**e**) or -thalamic (**f**) circuits during retrieval. The number of mice is indicated in the figure. Data are presented as mean± S.E.M.


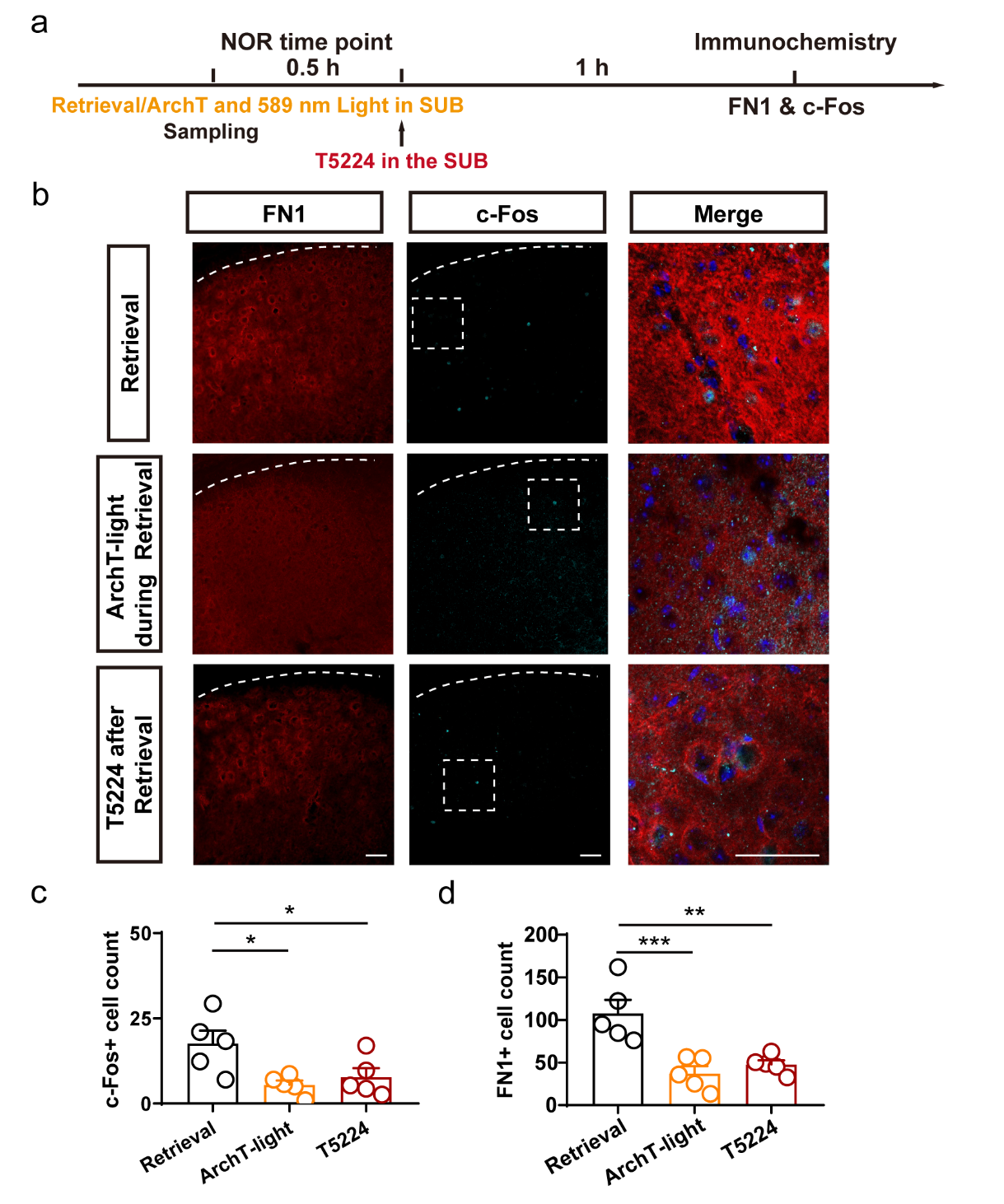


**Supplementary Figure 8. FN1 expression is activity-dependently regulated in NOR. a** Schematic of sampling for immunochemistry after different manipulation of novel object recognition (NOR) retrieval. **b** Representative images of FN1 expression (red) and co-labeled with c-Fos (cyan) after different manipulation of NOR retrieval. Scale bar, 50 μm. **c** Quantification of c-Fos+ (c) and FN1+ (d) cells. One-way ANOVA with *post hoc* Dunnett’s multiple comparisons. **p*<0.05, ***p*<0.01, ****p*<0.001. N = 5, 5, 5 mice. Data are presented as mean± S.E.M.


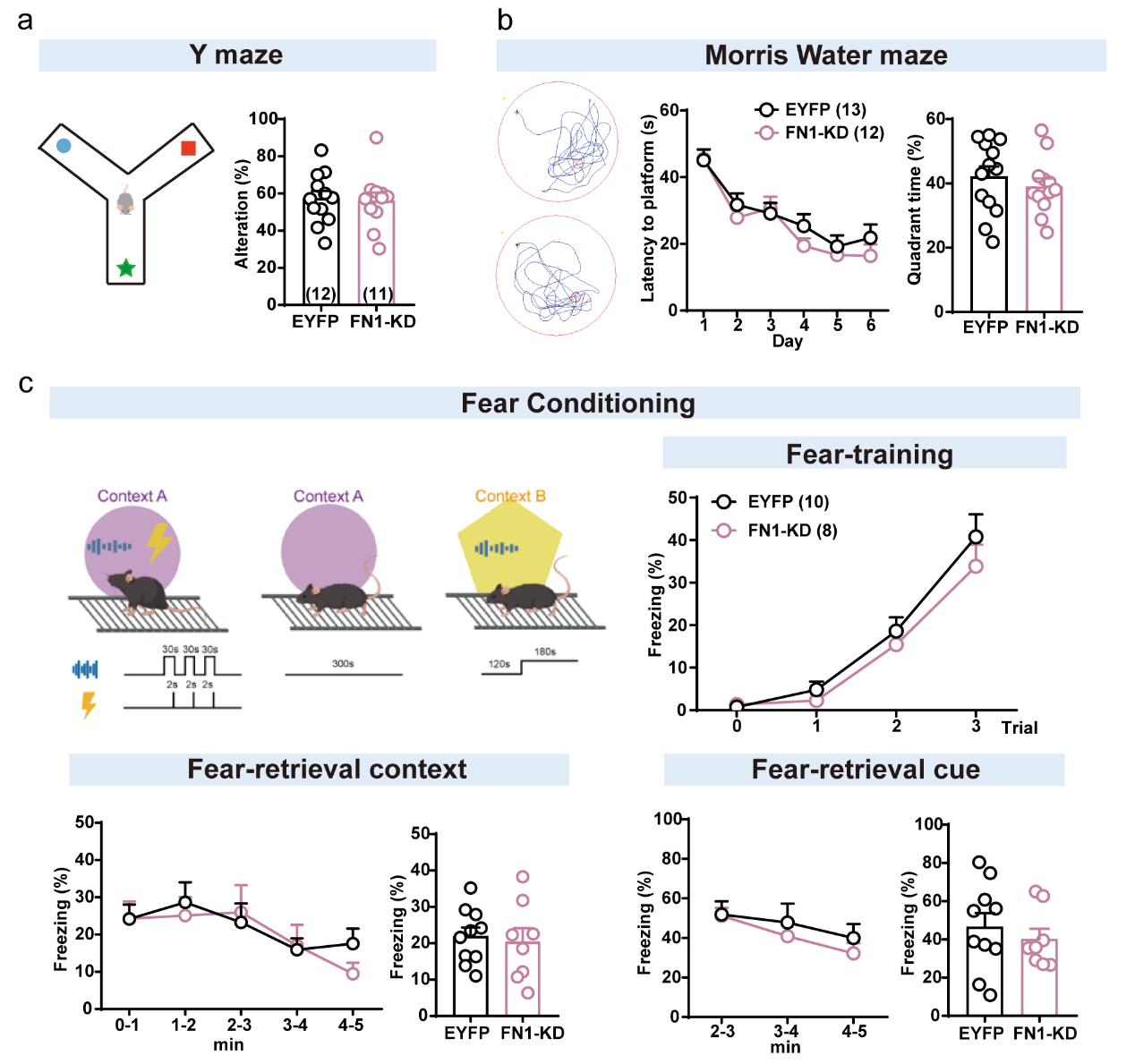


**Supplementary Figure 9.** **FN1 knockdown does not affect spatial memory and conditional fear memory. a** Alteration in Y maze by subicular FN1 knockdown (KD). **b** Latency to platform in Morris water maze during training by subicular FN1-KD. **c** Freezing ratio in conditional fear memory by subicular FN1-KD. The number of mice is indicated in the figure. Data are presented as mean± S.E.M.


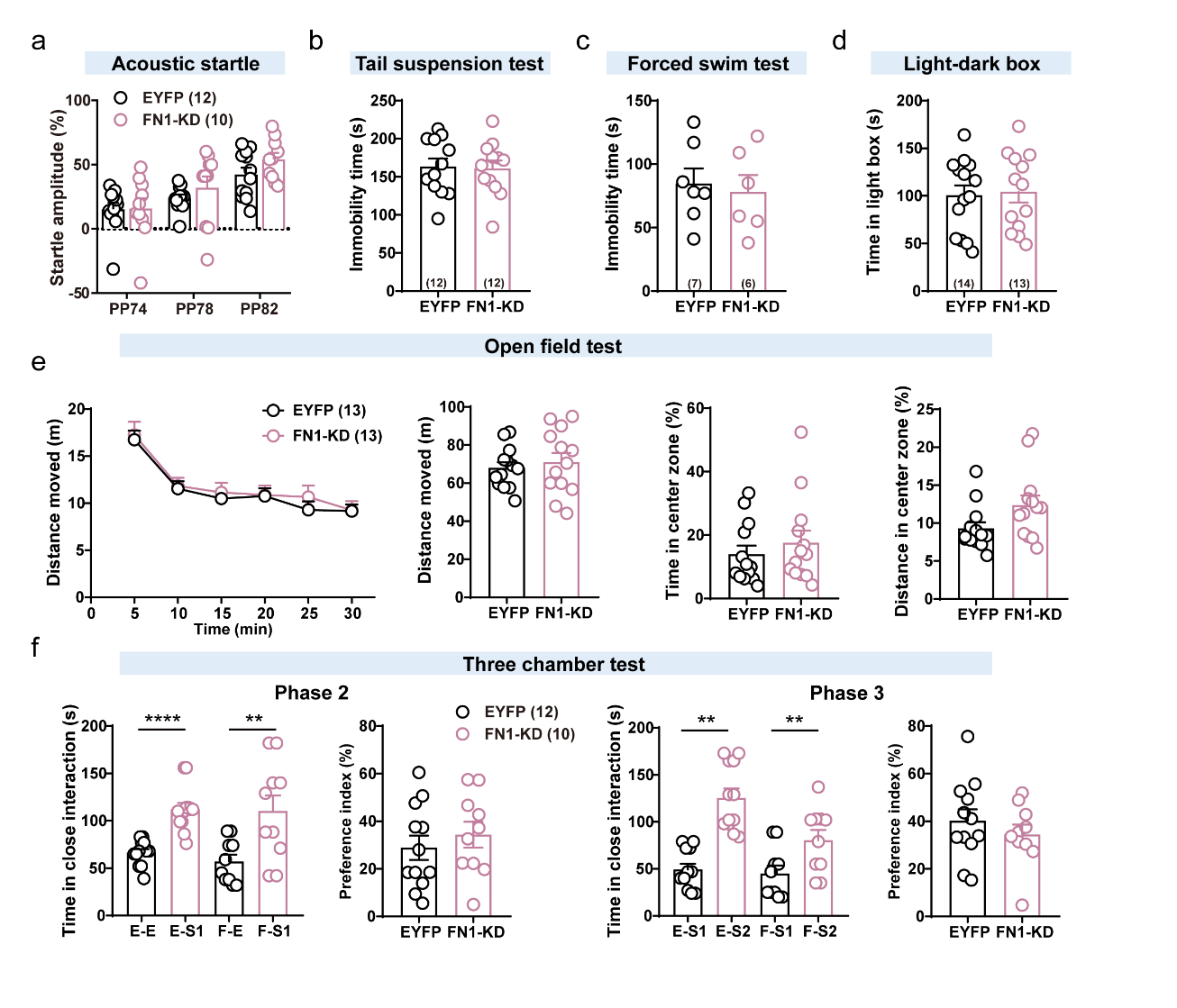


**Supplementary Figure 10. FN1 knockdown does not affect emotion-related behaviors. a** Startle amplitude induce by different decibels of sound in acoustic startle by subicular FN1 knockdown (KD). **b** Immobility time in tail suspension test by subicular FN1-KD. **c** Immobility time in forced swim test by subicular FN1-KD. **d** Time in light box during light-dark box test by subicular FN1-KD. **e** Distance moved, time in center zone (%) and distance in center zone (%) during light-dark box test by subicular FN1-KD. **f** Time in close interaction and preference index (%) during three-chamber test by subicular FN1-KD. The number of mice is indicated in the figure. Data are presented as mean± S.E.M.

**
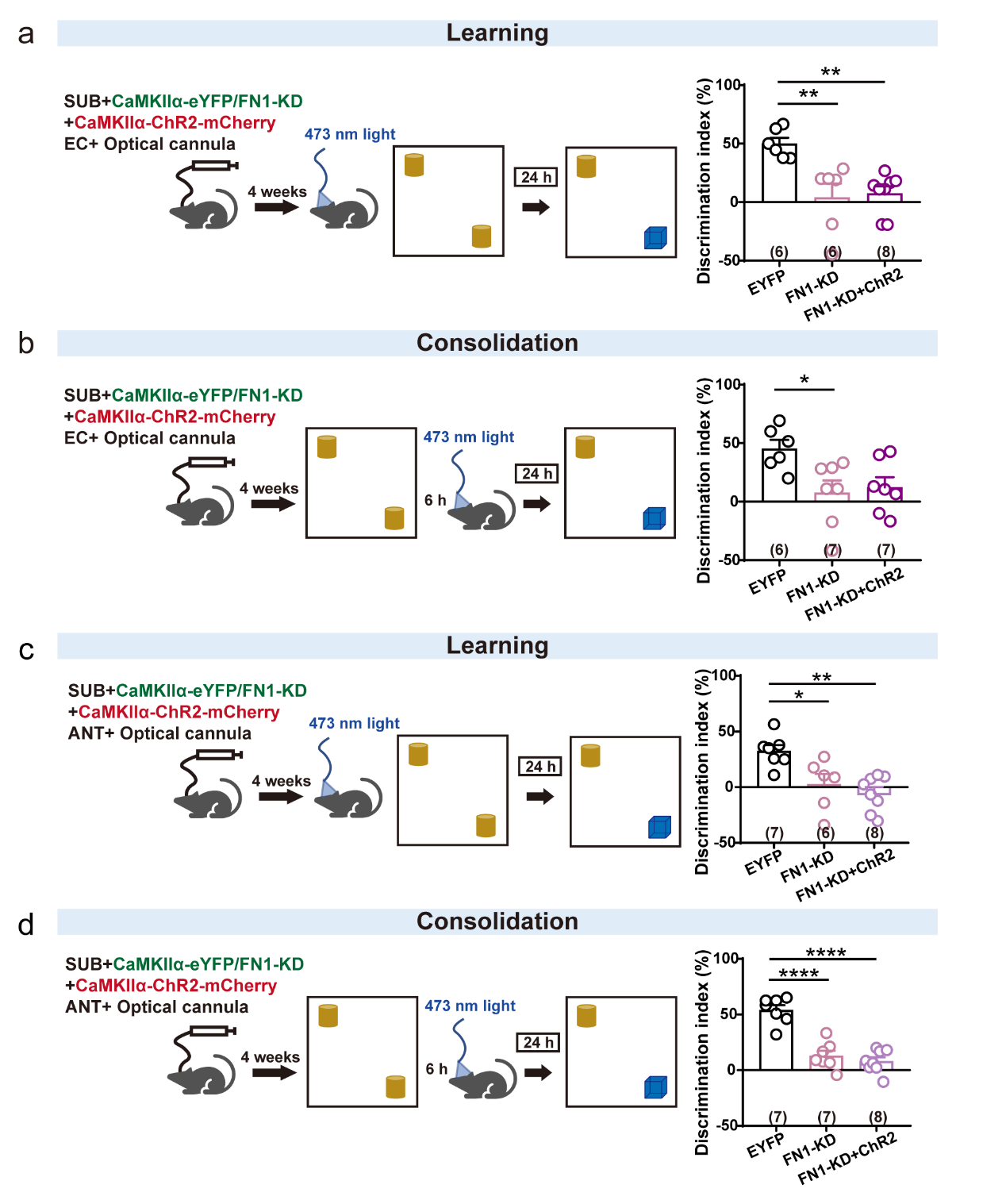
**

**Supplementary Figure 11. Activation of subicular-entorhinal and -thalamic circuits during learning or consolidation period cannot reverse the FN1 knockdown-induced NOR impairment. a** and **b** Discrimination index in novel object recognition (NOR) test by subicular FN1 knockdown and opto-activation of subicular-entorhinal circuit during learning (**a**) or consolidation (**b**) period. **c** and **d** Discrimination index in NOR test by subicular FN1 knockdown and opto-activation of subicular-entorhinal circuit during learning (**c**) or consolidation (**d**) period. One-way ANOVA with *post hoc* Tukey’s multiple comparisons, **p*<0.05, ***p*<0.01, *****p*<0.0001. The number of mice is indicated in the figure. Data are presented as mean± S.E.M.

**
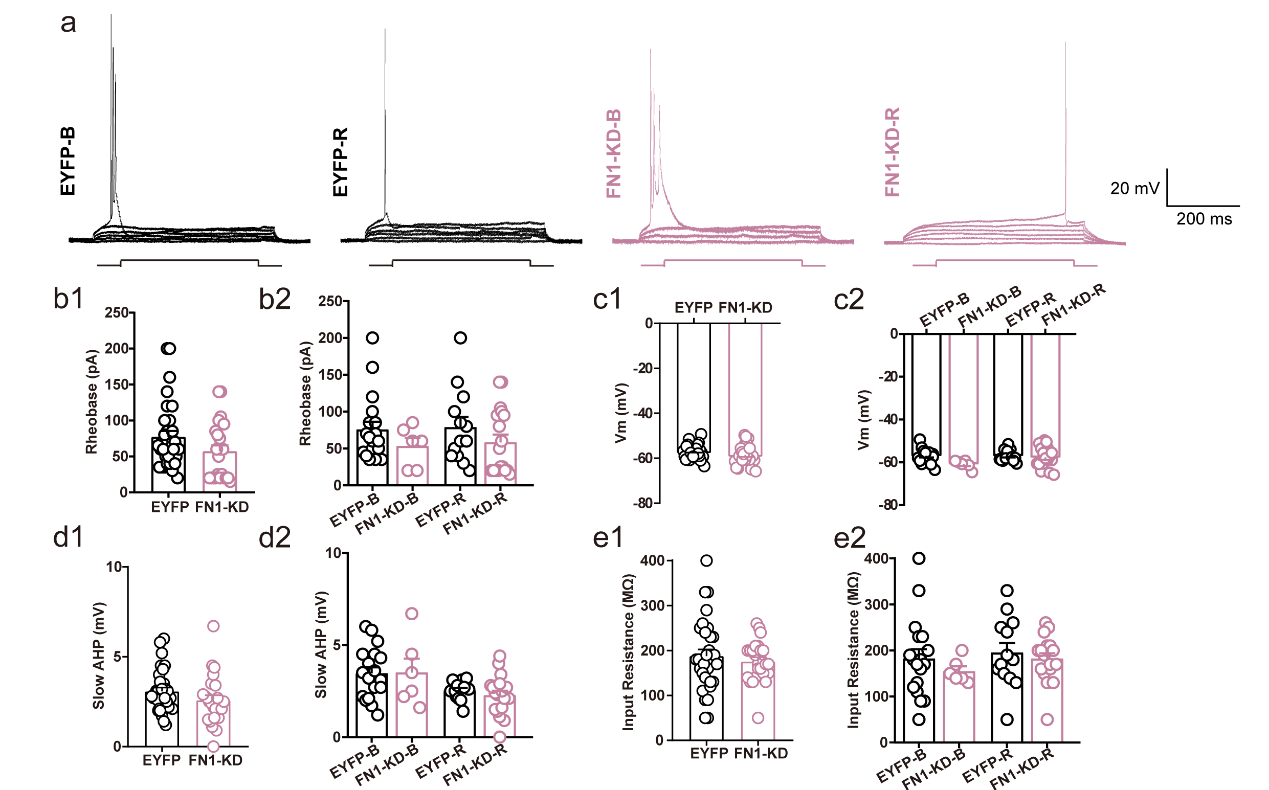
**

**Supplementary Figure 12. Electrophysical properties of subicular pyramidal neurons by FN1 knockdown. a** Representative traces of subicular bursting and regular firing cells evoked by gradients of depolarization currents by FN1 knockdown (KD). Each step represented 20 pA, until cells elicited first AP. **b-e** Rheobase (**b**), resting membrane potential (Vm, **c**), slow AHP size (**d**) and input resistance **(e)** of subicular bursting and regular spiking cells by FN1-KD. Data are presented as mean± S.E.M.


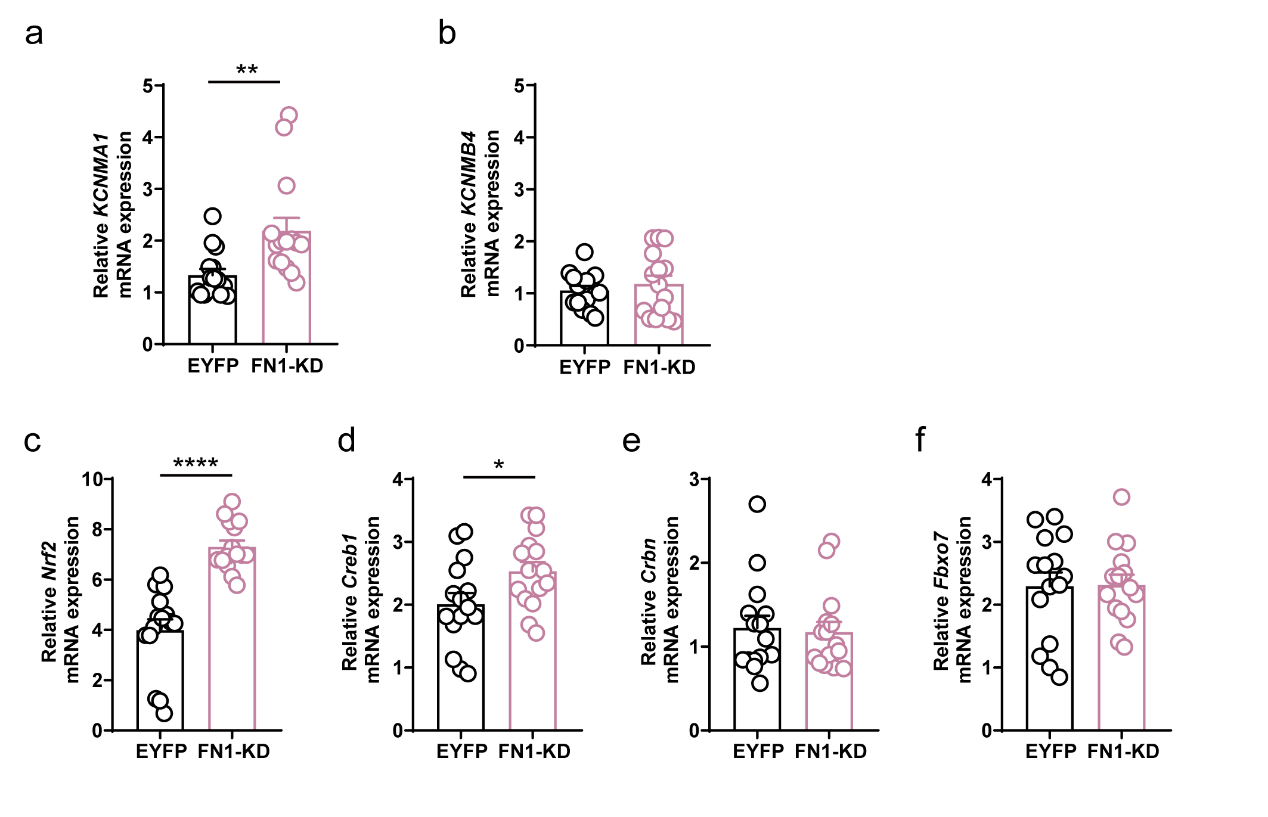


**Supplementary Figure 13. Relative mRNA expression in the subiculum by FN1 knockdown. a** and **b** Relative *KCNMA1* (**a**) and *KCNMB4* (**b**) mRNA expression of by FN1-KD. Unpaired *t*-test, ***p*<0.01. **c-f** Relative *Nrf2* (**c**), *Creb1* (**d**), *Crbn* (**e**) and *Fbox7* (**f**) mRNA expression of by FN1-KD. Unpaired *t*-test, **p*<0.05; *****p*<0.0001. N **=** 5 mice, each mouse including 3 samples. Data are presented as mean± S.E.M.

**Supplementary Table 1. Total exploration times during NOR and OLR tests.**

| **Figure** | **Duration (Mean ± SEM, s)** | | | | | | | |
| --- | --- | --- | --- | --- | --- | --- | --- | --- |
|  | mCherry-new | mCherry-old | hM3Dq-new | hM3Dq-old | hM4Di-new | hM4Di-old |  |  |
| 1m | 8.500 ± 1.150 | 5.582 ± 1.027 | 7.638 ± 1.266 | 5.646 ± 1.578 | 6.764 ± 1.040 | 5.700 ± 1.168 |  |  |
| 1n | 7.440 ± 0.853 | 4.530 ± 0.768 | 8.300 ± 1.118 | 4.378 ± 0.557 | 7.583 ± 1.686 | 5.908 ± 1.694 |  |  |
| 1o | 9.129 ± 0.850 | 6.193 ± 0.713 | 10.66 ± 1.116 | 5.033 ± 0.565 | 6.933 ± 1.142 | 6.467 ± 0.874 |  |  |
|  | | | | | | | | |
|  | eyfp-new | eyfp-old | Chr2-new | Chr2-old | eyfp-new | eyfp-old | Arch-new | Arch-old |
| 2f-g | 7.140 ± 1.001 | 3.180 ± 0.470 | 6.37 ± 0.948 | 2.330 ± 0.263 | 7.056 ± 1.128 | 3.356 ± 0.409 | 6.591 ± 1.372 | 4.009 ± 0.610 |
| 2i-j | 7.500 ± 1.428 | 3.367 ± 0.538 | 7.609 ± 1.446 | 3.909 ± 0.598 | 8.691 ± 1.405 | 4.300 ± 0.799 | 11.29 ± 2.448 | 6.100 ± 1.352 |
| 2l-m | 5.145 ± 0.661 | 2.718 ± 0.399 | 11.16 ± 1.730 | 3.427 ± 0.709 | 6.125 ± 1.437 | 2.650 ± 0.547 | 3.811 ± 0.712 | 2.633 ± 0.577 |
|  | eyfp-new | eyfp-old | Chr2-new | Chr2-old | eyfp-new | eyfp-old | Chr2-new | Chr2-old |
| 2o-p | 9.609 ± 2.170 | 4.591 ± 1.189 | 8.585 ± 1.584 | 5.546 ± 0.685 | 9.880 ± 1.995 | 4.710 ± 0.616 | 9.869 ± 1.408 | 5.046 ± 0.524 |
|  | | | | | | | | |
|  | eyfp-new | eyfp-old | KD-new | KD-old | eyfp-new | eyfp-old | KD-new | KD-old |
| 3k-l | 17.25 ± 2.955 | 5.771 ± 1.035 | 13.86 ± 2.320 | 11.82 ± 2.166 | 15.38 ± 2.636 | 10.1 ± 1.962 | 11.76 ± 1.702 | 6.964 ± 1.106 |
|  | | | | | | | | |
|  | eyfp-new | eyfp-old | KD-new | KD-old | KD+ChR2-new | KD+ChR2-old |  |  |
| 4j | 8.814 ± 1.575 | 4.071 ± 0.852 | 6.283 ± 1.629 | 7.317 ± 2.889 | 9.275 ± 2.104 | 4.113 ± 0.732 |  |  |
| 4k | 8.914 ± 2.121 | 4.143 ± 1.145 | 3.843 ± 0.701 | 4.143 ± 0.651 | 4.429 ± 0.778 | 4.371 ± 0.687 |  |  |
|  | | | | | | | | |
|  | eyfp-new | eyfp-old | paxi-new | paxi-old | KD-new | KD-old | KD-paxi-new | KD-paxi-old |
| 5n | 9.456 ± 0.679 | 5.178 ± 0.594 | 9.600 ± 1.481 | 3.511 ± 0.548 | 6.286 ± 0.735 | 6.229 ± 0.889 | 7.289 ± 0.822 | 4.356 ± 0.679 |
|  | | | | | | | | |
|  | mCherry-new | | mCherry-old | | hM4Di-new | | hM4Di-old | |
| S2c | 13.89 ± 1.532 | | 7.567 ± 1.044 | | 8.579 ± 0.917 | | 5.671 ± 0.697 | |
|  | | | | | | | | |
|  | eyfp-new | eyfp-old | Chr2-new | Chr2-old | eyfp-new | eyfp-old | Chr2-new | Chr2-old |
| S5b-c | 9.427 ± 1.508 | 5.318 ± 1.048 | 11.58 ± 1.502 | 4.629 ± 0.693 | 12.60 ± 3.049 | 4.725 ± 1.160 | 14.66 ± 2.362 | 5.443 ± 0.860 |
| S5e-f | 9.420 ± 1.928 | 4.490 ± 1.042 | 10.23 ± 1.266 | 4.700 ± 0.657 | 12.03 ± 1.763 | 5.845 ± 0.824 | 16.91 ± 2.079 | 7.871 ± 1.040 |
|  | eyfp-new | eyfp-old | Arch-new | Arch-old | eyfp-new | eyfp-old | Arch-new | Arch-old |
| S5h-i | 13.53 ± 1.869 | 7.120 ± 1.421 | 14.39 ± 1.851 | 6.700 ± 0.866 | 15.22 ± 2.421 | 6.817 ± 1.133 | 16.66 ± 4.463 | 7.364 ± 1.379 |
| S5k-l | 9.564 ± 2.085 | 4.836 ± 1.269 | 11.92 ± 1.185 | 7.631 ± 0.990 | 14.32 ± 2.299 | 7.464 ± 1.241 | 18.52 ± 3.260 | 7.408 ± 0.960 |
| S5n-o | 5.567 ± 1.566 | 3.083 ± 0.759 | 9.218 ± 2.26 | 3.955 ± 0.748 | 8.836 ± 1.370 | 4.945 ± 0.751 | 12.58 ± 1.321 | 6.021 ± 0.941 |
|  | | | | | | | | |
|  | eyfp-new | | eyfp-old | | Arch-new | | Arch-old | |
| S6f | 9.867 ± 1.994 | | 5.233 ± 1.676 | | 10.18 ± 1.327 | | 7.792 ± 1.921 | |
| S6h | 9.733 ± 2.375 | | 4.867 ± 0.724 | | 8.230 ± 1.265 | | 6.290 ± 1.376 | |
| S6j | 9.400 ± 0.979 | | 4.850 ± 0.857 | | 6.989 ± 1.089 | | 5.267 ± 1.321 | |
|  | | | | | | | | |
|  | eyfp-new | eyfp-old | Chr2-new | Chr2-old | eyfp-new | eyfp-old | Chr2-new | Chr2-old |
| S7c-d | 6.125 ± 1.085 | 4.008 ± 0.979 | 6.491 ± 1.255 | 4.164 ± 1.016 | 6.180 ± 0.633 | 3.650 ± 0.783 | 8.282 ± 0.923 | 5.518 ± 0.924 |
|  | eyfp-new | eyfp-old | Arch-new | Arch-old | eyfp-new | eyfp-old | Arch-new | Arch-old |
| S7e-f | 6.277 ± 0.983 | 3.592 ± 0.729 | 4.845 ± 0.955 | 2.400 ± 0.302 | 7.525 ± 1.370 | 4.008 ± 0.833 | 7.309 ± 1.387 | 4.227 ± 0.957 |
|  | | | | | | | | |
|  | eyfp-new | eyfp-old | KD-new | KD-old | KD+ChR2-new | KD+ChR2-old |  |  |
| S11a | 7.083 ± 1.452 | 2.550 ± 0.574 | 2.957 ± 0.779 | 3.771 ± 2.214 | 3.843 ± 1.767 | 2.286 ± 0.661 |  |  |
| S111b | 5.650 ± 1.288 | 2.183 ± 0.657 | 1.950 ± 0.362 | 1.750 ± 0.385 | 2.863 ± 0.648 | 2.575 ± 0.619 |  |  |
| S11c | 11.31 ± 3.390 | 6.300 ± 1.933 | 5.517 ± 1.038 | 5.283 ± 0.841 | 6.488 ± 0.732 | 7.275 ± 0.748 |  |  |
| S11d | 9.043 ± 2.165 | 2.700 ± 0.619 | 5.914 ± 0.572 | 4.614 ± 0.601 | 4.663 ± 0.636 | 4.050 ± 0.601 |  |  |

**Supplementary Table 2. Statistical details for main figures.**

| **Figure** | **Statistics** | ***p*** |
| --- | --- | --- |
| 1f j | Paired *t* test, f: *t*=0.005282, df=13; j: *t*=3.615, df=13 | f: 0.9959, j: 0.0031 |
| 1m | One-Way ANOVA with Dunnett's, *F* (2, 32) = 1.243 | mCherry vs. hM3Dq 0.8621  mCherry vs. hM4Di 0.2326 |
| 1n | One-Way ANOVA with Dunnett's, *F* (2, 28) = 2.026 | mCherry vs. hM3Dq 0.9121  mCherry vs. hM4Di 0.2350 |
| 1o | One-Way ANOVA with Dunnett's, *F* (2, 29) = 10.72 | mCherry vs. hM3Dq 0.0448  mCherry vs. hM4Di 0.0211 |
| 2f-g | Unpaired *t* test, f: *t*=0.4783, df=18; g: *t*=1.668, df=18 | f: 0.6382, g: 0.1126 |
| 2i-j | Unpaired *t* test, i: *t*=0.4648, df=18; j: *t*=0.3605, df=21 | i: 0.6476, j: 0.7220 |
| 2l-m | Unpaired *t* test, l: *t*=3.573, df=20; m: *t*=3.063, df=15 | l: 0.0019, m: 0.0079 |
| 2o-p | Unpaired *t* test, o: *t*=2.315, df=22; p: *t*=0.05021, df=21 | o: 0.0303, p: 0.9604 |
| 3b | Unpaired *t* test, t=17.16, df=6 | <0.0001 |
| 3e | One-Way ANOVA with Tukey's, F (3, 16) = 10.01 | Sham vs. Retrieval 0.0030 |
| 3f | One-Way ANOVA with Tukey's, F (3, 16) = 10.62 | Sham vs. Retrieval 0.0002 |
| 3g | One-Way ANOVA with Tukey's, F (3, 16) = 18.70 | Sham vs. Learning 0.0116 Sham vs. Consolidation 0.023 Sham vs. Retrieval <0.0001 |
| 3j | Unpaired *t* test, t=3.237, df=4 | 0.0318 |
| 3k l | Unpaired *t* test, k: *t*=5.400, df=25; l: *t*=0.1995, df=19 | k: <0.0001, l: 0.8440 |
| 4d h | Paired *t* test, d: *t*=0.4348, df=11; h: *t*=0.7341, df=11 | d: 0.6721, h: 0.4782 |
| 4j | One-Way ANOVA with Tukey's, *F* (2, 18) = 4.770 | EYFP vs. KD 0.0287  EYFP vs. KD+ChR2 0.9446  KD vs. KD+ChR2 0.0451 |
| 4k | One-Way ANOVA with Tukey's, *F* (2, 18) = 7.749 | EYFP vs. KD 0.0041  EYFP vs. KD+ChR2 0.0227  KD vs. KD+ChR2 0.7088 |
| 5b | Two-Way ANOVA, *F* (1, 1113) = 9.026 | 0.0027 |
| 5d-g | Unpaired *t* test, d: *t*=1.245, df=53; *e*: t=3.840, df=53, *f*: t=1.281, df=53; *g*: t=4.491, df=53 | d: 0.2186, e: 0.0003, f: 0.2056, g: <0.0001 |
| 5h | Fisher's exact test | 0.0273 |
| 5i | Two-Way ANOVA with Tukey's, *F* (1, 51) = 39.48 | B: EYFP vs. B: KD 0.0001  R:EYFP vs. R: KD 0.0006 |
| 5j | Two-Way ANOVA with Tukey's, *F* (1, 51) = 0.4969 | B: EYFP vs. B: KD 0.9979  R: EYFP vs. R: KD 0.5127 |
| 5k | Two-Way ANOVA with Tukey's, *F* (1, 51) = 9.253 | B: EYFP vs. B: KD 0.9890  R: EYFP vs. R: KD 0.0002 |
| 5m | Unpaired *t* test, *t*=8.324, df=8 | <0.0001 |
| 5n | One-Way ANOVA with Tukey's, *F* (3, 30) = 13.19 | Sham vs. Paxilline 0.0806  Sham vs. KD 0.0025 KD vs. KD+Paxilline 0.0094 |
| 6c | Two-Way ANOVA with Tukey's, *F* (4, 15) = 12.27 | Anterior SUB ＞0.9999,  Posterior SUB <0.0001, CA1 0.0003, DG 0.8367, PrS 0.0005 |
| 6i | Paired *t* test, *t*=0.8105, df=11 | 0.4348 |

**Supplementary Table 3. Statistical details for supplementary figures.**

| **Figure** | **Statistics** | ***p*** |
| --- | --- | --- |
| S2c | Unpaired *t* test, *t*=1.427, df=24 | 0.1664 |
| S4 | Friedman with Dunn's, Friedman statistic=12.07 | Light vs. Pre, 0.0017;  Light vs. Post, 0.5701 |
| S5b-c | Unpaired *t* test, b: *t*=1.524, df=23; c: *t*=0.2145, df=20 | b: 0.1412, c: 0.8323 |
| S5e-f | Unpaired *t* test, e: *t*=0.5287, df=22; f: *t*=0.3924, df=24 | e: 0.6023, f: 0.6982 |
| S5h-i | Unpaired *t* test, h: *t*=0.2117, df=21; i: *t*=0.6708, df=23 | h: 0.8344, i: 0.5090 |
| S5k-l | Unpaired *t* test, k: *t*=0.8409, df=22; l: *t*=1.249, df=21 | k: 0.4095, l: 0.2256 |
| S5n-o | Unpaired *t* test, n: *t*=1.290, df=15; o: *t*=0.9536, df=23 | n: 0.2166, o: 0.3502 |
| S6f | Unpaired *t* test, *t*=1.122, df=16 | 0.2785 |
| S6h | Unpaired *t* test, *t*=0.5433, df=14 | 0.5954 |
| S6j | Unpaired *t* test, *t*=1.295, df=15 | 0.2148 |
| S7c-d | Unpaired *t* test, c: *t*=0.3742, df=21; d: *t*=0.6845, df=19 | c: 0.7120, d: 0.5020 |
| S7e-f | Unpaired *t* test, e: *t*=0.1424, df=22; f: *t*=0.8855, df=21 | e: 0.8881, f: 0.3859 |
| S8c | One-Way ANOVA with Dunnett's, F (2, 12) = 5.463 | Retrieval vs. ArchT-light: 0.0165;  Retrieval vs. T5224: 0.0488 |
| S8d | One-Way ANOVA with Dunnett's, F (2, 12) = 12.90 | Retrieval vs. ArchT-light: 0.0010;  Retrieval vs. T5224: 0.0033 |
| S9a | Unpaired *t* test, *t*=0.2272, df=21 | 0.8225 |
| S9b | Two-way ANOVA, F (1, 138) = 2.035;  Unpaired *t* test, *t*=0.7691, df=23 | 0.156;  0.4497 |
| S9c | Unpaired t test, context: *t*=0.3684, df=16;  cue: *t*=0.6697, df=16 | Context: 0.7174; Cue: 0.5126 |
| S10a | Two-way ANOVA, F (1, 60) = 1.917 | 0.1713 |
| S10b | Unpaired *t* test, *t*=0.1506, df=22 | 0.8817 |
| S10c | Unpaired *t* test, *t*=0.3751, df=11 | 0.7147 |
| S10d | Unpaired *t* test, *t*=0.2303, df=25 | 0.8198 |
| S10e | Two-way ANOVA, F (1, 144) = 0.8754 | 0.351 |
| S10f | Unpaired *t* test, 2: *t*=0.7334, df=20; 3: *t*=0.8754, df=20 | 2-0.4718, 3-0.3918 |
| S11a | One-Way ANOVA with Tukey's, F (2, 17) = 9.825 | EYFP vs. KD 0.0031  EYFP vs. KD+ChR2 0.0034  KD vs. KD+ChR2 0.9484 |
| S11b | One-Way ANOVA with Tukey's, F (2, 17) = 4.829 | EYFP vs. KD 0.0264  EYFP vs. KD+ChR2 0.0538  KD vs. KD+ChR2 0.9273 |
| S11c | One-Way ANOVA with Tukey's, F (2, 18) = 9.361 | EYFP vs. KD 0.0180  EYFP vs. KD+ChR2 0.0016  KD vs. KD+ChR2 0.6705 |
| S11d | One-Way ANOVA with Tukey's, F (2, 19) = 35.58 | EYFP vs. KD <0.0001  EYFP vs. KD+ChR2 <0.0001  KD vs. KD+ChR2 0.6883 |
| S12b2 | Two-way ANOVA, F (1, 51) = 2.591 | 0.1136 |
| S12c2 | Two-way ANOVA, F (1, 51) = 4.681 | 0.0352 |
| S12d2 | Two-way ANOVA, F (1, 51) = 0.09846 | 0.755 |
| S12e2 | Two-way ANOVA, F (1, 51) = 0.9759 | 0.3279 |
| S13a | Unpaired *t* test, *t*=3.112, df=28 | 0.0042 |
| S13b | Unpaired *t* test, *t*=0.6893, df=28 | 0.4963 |
| S13c | Unpaired *t* test, *t*=6.632, df=28 | <0.0001 |
| S13d | Unpaired *t* test, *t*=2.237, df=28 | 0.0334 |
| S13e | Unpaired *t* test, *t*=0.7920, df=28 | 0.2662 |
| S13f | Unpaired *t* test, *t*=0.07208, df=28 | 0.9430 |
